# Integration of proteomic and transcriptomic profiles reveals multiple levels of genetic regulation of salt tolerance in cotton

**DOI:** 10.1186/s12870-018-1350-1

**Published:** 2018-06-20

**Authors:** Zhen Peng, Shoupu He, Wenfang Gong, Feifei Xu, Zhaoe Pan, Yinhua Jia, Xiaoli Geng, Xiongming Du

**Affiliations:** State Key Laboratory of Cotton Biology, Institute of Cotton Research, Chinese Academy of Agricultural Sciences, Anyang, 455000 Henan China

**Keywords:** Salt stress, Proteomics, Transcriptome, Alternative splicing, miRNAs

## Abstract

**Background:**

Salinity is a major abiotic stress that limits upland cotton growth and reduces fibre production worldwide. To reveal genetic regulation via transcript and protein levels after salt stress, we comprehensively analysed the global changes in mRNA, miRNA, and protein profiles in response to salt stress in two contrasting salt-tolerant cotton genotypes.

**Results:**

In the current study, proteomic and mRNA-seq data were combined to reveal that some genes are differentially expressed at both the proteomic and mRNA levels. However, we observed no significant change in mRNA corresponding to most of the strongly differentially abundant proteins. This finding may have resulted from global changes in alternative splicing events and miRNA levels under salt stress conditions. Evidence was provided indicating that several salt stress-responsive proteins can alter miRNAs and modulate alternative splicing events in upland cotton. The results of the stringent screening of the mRNA-seq and proteomic data between the salt-tolerant and salt-sensitive genotypes identified 63 and 85 candidate genes/proteins related to salt tolerance after 4 and 24 h of salt stress, respectively, between the tolerant and sensitive genotype. Finally, we predicted an interaction network comprising 158 genes/proteins and then discovered that two main clusters in the network were composed of ATP synthase (CotAD_74681) and cytochrome oxidase (CotAD_46197) in mitochondria. The results revealed that mitochondria, as important organelles involved in energy metabolism, play an essential role in the synthesis of resistance proteins during the process of salt exposure.

**Conclusion:**

We provided a plausible schematic for the systematic salt tolerance model; this schematic reveals multiple levels of gene regulation in response to salt stress in cotton and provides a list of salt tolerance-related genes/proteins. The information here will facilitate candidate gene discovery and molecular marker development for salt tolerance breeding in cotton.

**Electronic supplementary material:**

The online version of this article (10.1186/s12870-018-1350-1) contains supplementary material, which is available to authorized users.

## Background

Increasingly, abiotic stresses such as salinity are severely threatening crop productivity and reducing the quality of plants worldwide [1]. Salinity can affect cellular osmotic and ionic homeostasis as well as photosynthesis; deplete cellular energy; and lead to redox imbalance, reduced growth, and even plant death [2, 3]. Plants must evolve effective strategies to adapt to various biotic and abiotic stresses by manoeuvring specialized gene expression programmes that promote stress protection, homeostasis, and survival [4–6]. During the recent decade, an increasing number of studies have focused on the molecular mechanisms of salt tolerance in model plants such as Arabidopsis and rice [1, 7, 8]. Understanding of the salt tolerance mechanisms of these model plants is undoubtedly of great significance for studying the salt tolerance mechanisms of those crops [9, 10].

Cotton (*Gossypium hirsutum* L.), which is a crucial cash crop, is the second most inherently salt-tolerant crop and is more tolerant than both rice (*Oryza sativa*) and *Arabidopsis thaliana* [11]. However, inhibited growth and reduced productivity occur when cotton plants are exposed to the high salinity conditions, especially when this exposure occurs at the germination or seedling stage [12, 13]. Moreover, as the cultivated land area diminishes, competition between grain crops and cotton is becoming increasingly prominent; as such, cotton cultivation has gradually moved to saline and alkaline lands [14]. Therefore, to breed salt-tolerant cotton cultivars, it is essential to clarify the salt tolerance mechanism of cotton and identify the salt tolerance-related genes. Unfortunately, the salt resistance of most cotton varieties was gradually lost due to selection for yield at the later stages of domestication [15]. Hence, to identify the salt tolerance genes/proteins, analysing genotypes with similar genetic backgrounds and contrasting salt tolerance is preferred. In fact, the salt response of the glycophyte *A. thaliana* was recently compared with that of the halophyte *Thellungiella salsuginea* [16–18]. Apart from these studies, other studies involving rice [19], wheat [20, 21], tomato [22], cotton [23–25], and soybean [26] have been conducted to understand the differences in gene/protein expression between salt-sensitive and salt-tolerant genotypes.

Despite extensive descriptions of the molecular regulatory pathways involved in salt stress tolerance, interpretations of genotype-related differences in the salt tolerance mechanisms of cotton have been facilitated by large-scale experimentation processes involving ‘omics’, such as transcriptomics [23, 24, 27–29] and proteomics [25, 30, 31]. The precise analysis of the proteome is essential for understanding underlying stress physiology and needs to be further elucidated at multiple levels.

Despite recent studies, complete description of the proteome is still challenging because of the complexity of mRNA splicing during transcription [32, 33]. In addition to transcriptional regulation, gene expression is also clearly modulated at the post-transcriptional level; for example, miRNAs can regulate specific genes that target mRNAs for degradation or inhibit translation [34, 35]. Regardless of the mechanism, since biological processes in response to salt stress are ultimately controlled by proteins, genome-wide proteomic analyses are crucial for providing accurate pictures of the regulatory networks of functional genes and proteins. However, we rarely understand how changes in transcriptional, post-transcriptional or even translational control are reflected in changes at the protein level. Poor correlations between mRNA and protein expression have often been reported [36, 37], although some recent reports have revealed a modest relationship between the regulation of the two levels [38, 39]. A recent study addressing the alterations in mRNA and protein abundance in upland cotton emphasized the importance of translational regulation of protein abundance [40], reflecting the significance of a comprehensive view of gene expression.

In our study, by exploiting isobaric tag for relative and absolute quantitation (iTRAQ)-based proteomics, which is both high throughput and quantitative, as well as the transcriptomic data of mRNAs, alternative splicing (AS), and miRNAs in the same samples, for the first time, we were able to analyse in depth the relationships among the gene expression of these important players and reveal the mechanisms of gene regulation during salt stress between the two contrasting genotypes. We compared the protein and mRNA expression levels and analysed the correlations between the differences in mRNA and protein abundance. The results indicated that the gene and protein expression profiles were weakly correlated, but some stress-responsive genes were regulated in accordance with the mRNA levels and translational efficiency, which was evident for both stress-induced and stress-repressed genes. Several genes showed the opposite trend, suggesting that antagonistic regulation occurs at the mRNA and translation levels. The results of our analysis also revealed different salt tolerance mechanisms, in that several novel aspects of the metabolic and stress response pathways differ in cotton, revealing that their relationships with AS events and miRNAs are regulated. This study provides new clues concerning the interrelationship between salt tolerance-related genes/proteins and energy metabolism within mitochondria.

## Methods

### Plant materials and salt stress treatments

Plants of two upland cotton (*G. hirsutum* L.) genotypes, the salt-tolerant Earlistaple 7 (E7) and the salt-sensitive Nan Dan Ba Di Da Hua (NH), were grown in hydroponic containers containing 1/2-strength Hoagland solution in a phytotron (KR-III; Henan China) (28/22 °C day/night temperature, 60–80% relative humidity, and a 14/10 h light/dark cycle under 450 μmol m^− 2^ s^− 1^ light intensity).

After being exposed to 200 mM NaCl solutions for 4 or 24 h, the leaves of the control and treated NH and E7 seedlings were harvested and directly submerged in liquid nitrogen, after which they were stored at − 80 °C until mRNA and protein extractions. More than six seedling leaves were sampled from each genotype/treatment. Three biological replicates of each sample were performed for proteomic analysis; among these replicates, one was also used for transcriptomic sequencing (mRNA-seq and small RNA-seq) as previously described by Peng et al. [23]. New batches of seedlings were planted and grown independently in another year under the same conditions as those described above. A more rigorous quality of seedling samples was collected for further validation of the transcriptomic data.

### Protein extraction, iTRAQ labelling and LC-ESI-mass spectrum (MS)/MS analysis

The total proteins of the seedlings subjected to 200 mM NaCl for 0, 4, or 24 h were isolated and purified as described previously [41, 42]. The total protein supernatants of 18 samples (i.e., 2 genotypes × 3 time points × 3 replicates) were transferred to a new tube and quantified using a 2-D Quant Kit (General Electric Company, USA). Three biological replicates and three technical replicates were performed to verify the protein quality and concentration. iTRAQ analysis was performed by BGI (Shenzhen, China).

The total protein (approximately 100 μg) content was removed from each sample solution, after which the protein was digested with trypsin. The peptides were subsequently dried by vacuum centrifugation. Peptides were reconstituted in 0.5 M tetraethylammonium bromide (TEAB) and labelled using an iTRAQ 8-plex kit (Applied Biosystems, Foster City, USA) in accordance with the manufacturer’s instructions. The NH peptides treated for 0 (control), 4, and 24 h (SN0, SN4, and SN24, respectively, for short) were labelled with iTRAQ tags 113,115, and 117, respectively, while the E7 peptides (SE0, SE4, and SE24, respectively, for short) were labelled with iTRAQ tags 114, 116, and 118, respectively. After they were labelled with the isobaric tags, the peptides were incubated at room temperature for 2 h. The peptide mixtures were then pooled and dried by vacuum centrifugation.

After the pooled peptides were labelled, they were dried and dissolved for fractionation by strong cation exchange (SCX) chromatography using an LC-20AB HPLC pump system (Shimadzu, Kyoto, Japan). The pooled peptides in the mixtures were reconstituted with 4 mL of buffer A (25 mM NaH_2_PO_4_ in 25% acetonitrile (ACN), pH 2.7). The SCX experiment was performed as described by Liu et al. [43] (2015). The peptides of each fraction were analysed by LC-ESI-MS/MS using a Triple TOF 5600 system and were subsequently analysed in accordance with previously described methods [44, 45].

### Proteome database search, quantification, and annotation

Protein identification and quantification were performed using the Mascot (version 2.3.02) search engine embedded in Proteome Discoverer (Matrix Science, Boston, MA). After the raw data were loaded, the spectra of 12 fractions were combined into one MGF (Mascot generic format) file, which was used to perform the query. To improve the accuracy of protein identification, only the unique peptides were used for quantification. The query parameters were set as follows: trypsin was chosen as the enzyme, with one missed cleavage allowed; fragment MS tolerance, ± 0.1 Da; peptide MS tolerance, ± 0.05 Da; variable modification, Gln → pyro-Glu (N-term Q), oxidation (Met), and iTRAQ8plex (Y); fixed modification, carbamidomethyl (C), iTRAQ8plex (N-term), and iTRAQ8plex (K). The queries were made against the predicted protein database (76,943 entries) of the *G. hirsutum* AD_1_ genome [46], which was downloaded from https://www.cottongen.org/data/download/genome_BGI_AD1.

To improve the accuracy of protein identification, a peptide confidence significance threshold of *P* < 0.05 (95% confidence) and an ion score or expected cutoff of less than 0.05 (95% confidence) were used in the identification. Each confident protein identification involves at least one unique peptide and a false discovery rate (FDR) of less than 0.05 against the reversed protein database. Proteins are considered significantly differentially abundant if they have been quantified as having at least one peptide among three biological triplicates. In addition, proteins that have average ratios greater than 1.2 or less than 0.833 as well as a *P*-value < 0.05 were considered differentially abundant proteins (DAPs) in subsequent analyses.

To assign possible annotations, all protein sequences were queried using BlastP (e-value < 10^− 5^) against the following protein databases: the Non-redundant (Nr) (in NCBI), SwissProt (UniProt), TrEMBL, Clusters of Orthologous Groups (COG) and Kyoto Encylopedia of Genes and Genomes (KEGG) databases. Gene Ontology (GO) functional annotations were obtained from the Nr database using the Blast2GO program [47].

### Transcriptome sequencing and AS analysis

All six of the cDNA (for mRNA-seq) libraries and all six of the small RNA libraries of raw data were downloaded from the NCBI Sequence Read Archive (http://www.ncbi.nlm.nih.gov/sra/) under accession SRP043419. Importantly, the transcriptomic and proteomic data originated from the same batch of samples. After the low-quality sequence reads were removed, the clean reads were mapped to the *G. hirsutum* AD_1_ genome and reference sequence using SOAPaligner/Soap2, with 95% minimum identity [48]. The gene expression in all six libraries was normalized to reads per kilobase of exon model per million mapped reads (RPKM) [49]. A gene was considered differentially expressed (DE) between two samples if its |log_2_ ratio| ≥ 1 and its FDR was less than 0.001. At the same time, TopHat (version 2.1.0) was used to predict the various types of prevalent AS events [50]. In addition, we defined the differentially expressed genes (DEGs) that underwent AS as alternatively spliced DEGs (AS-DEGs).

After the clean reads were obtained from the small RNA sequencing raw reads and summarized, the remaining unique RNAs (ranging from 18 to 30 nt) were mapped to the upland cotton genome (AD_1_) sequence to analyse their expression and distribution in the reference sequences. Those sequences with a perfect match were retained for further analysis in accordance with standard bioinformatics criteria [51]. The remaining unique small RNA sequences were first queried via BLASTn against the *G. hirsutum* miRNAs in a miRBase database (miRBase 21.0; www.mirbase.org) to identify conserved miRNAs with no mismatches allowed. The remains of the mapping sequences were then used to predict potential novel miRNAs as described by Zhang et al. [52] (2013). In addition, the miRNAs with at least one sample read per million (RPM) ≥ 10 between two comparisons and those whose RPM sum of 6 samples was more than 60 were considered for differential expression analysis. Based on the criteria of a *P*-value < 0.05 and an absolute value of the log_2_-fold change > 1, differential miRNA expression was determined using DEGseq [53]. The targets of DE miRNAs were predicted by the web tool psRNATarget (http://plantgrn.noble.org/psRNATarget/) using the BGI *G. hirsutum* (version 1.0) coding DNA sequences (CDSs) as the library for the target search. Sequences having less than 3-nt mismatches with the query miRNA sequences were selected.

### Identification of DE salt tolerance-related genes/proteins

To further analyse the specificity of the DE salt tolerance-related genes/proteins between both genotypes at the two time points, we developed the following stringent screening parameters: (i) “DEGs&protein ns” and “diff.same” types commonly up- or down-regulated between NH and E7 (i.e., DEGs with |log_2_E4/E0| - |log_2_N4/N0| ≥ 0.5); (ii) “DEGs&protein ns”, with opposite responses to salt (i.e., “DEGs&protein ns” with log_2_E4/E0 ≥ 1 and log_2_N4/N0 ≤ − 1 or vice versa); (iii) “DEGs&protein ns” and “diff.same” types specifically up- or down-regulated only in the salt-tolerant genotype (E7); (iv) “DAP&gene ns” commonly up- or down-regulated between NH and E7 (i.e., DAPs with | Quantitation (E4/E0)| > |Quantitation (N4/N0)|); and (v) “DAPs&gene ns” types specifically up- or down-regulated only in the salt-tolerant genotype (E7). We also analysed the specificity of the differentially abundant salt tolerance-related proteins in which the proteins and their corresponding gene data differed in our mRNA-seq data between both genotypes at the two time points, and the salt tolerance-related proteins that were specifically up- or down-regulated only in the salt-tolerant genotype (E7) after 4 h and 24 h were used as selective conditions.

### Expression patterns of the protein–protein interaction (PPI) network

To investigate the interaction network of these proteins in cotton plants under salt stress, an interaction network of salt tolerance-related genes/proteins was constructed. First, the identified protein IDs were queried against homologous genes in Arabidopsis using The Arabidopsis Information Resource (TAIR) 10 database. The PPI data for Arabidopsis were then retrieved from the STRING database (http://string-db.org) [54]. The salt tolerance-related proteins were ultimately mapped to the PPI network, after which the Cytoscape tool (version 3.5.0; http://www.cytoscape.org/download.php) was used to visualize the network.

### Quantitative reverse transcription-PCR (qRT-PCR) analysis of miRNAs and target genes

By using the stem-loop qRT-PCR method to assay miRNA expression [55], we selected 17 miRNAs (12 known miRNAs and 5 novel miRNAs) to validate the miRNA expression of small RNA sequences. In addition, their corresponding target genes (20 DEGs) were also used to validate the DEG expression identified in the mRNA-seq data by qRT-PCR. The leaves from seedlings treated with 200 mM NaCl for 4 and 24 h were used for the above experiments. First-strand cDNA was synthesized using a PrimeScript™ RT reagent kit with gDNA eraser (TaKaRa, Japan) in accordance with the manufacturer’s instructions. The miRNA and gene primers used are listed in Additional file 1. The stem-loop qRT-PCR and qRT-PCR experiments were performed and analysed in accordance with previously described methods [23].

### Validating miRNA/target interactions using PAREsnip

To validate miRNA/target interactions, the PAREsnip program was used to analyse the following data sets: the DE miRNA of four comparisons (E4/E0, E24/E0, N4/N0, and N24/N0); degradome sequencing data sets; and the CDSs of *G. hirsutum* genes. The two released cotton degradome sequencing data sets (GSM1008997 (seedlings) and GSM1008999 (hypocotyl)) of upland cotton were retrieved from the NCBI database. For each comparison analysis, the following parameters were set: a maximum of 4.0 mismatches, 100 dinucleotide shuffles and a *P-*value < 0.05.

### Bioinformatics and statistical analysis

A functional enrichment analysis of the DEGs and DE proteins was performed to identify significantly overrepresented GO terms and KEGG pathways using DAVID version 6.7 [56]. Protein patterns and their corresponding gene profiles were clustered using Genesis (http://genome.tugraz.at/genesisclient/genesisclient_download.shtml) based on the K-means method [57]. Scatterplots and Pearson correlation coefficients (r values) between proteins and mRNA expression ratios were constructed and calculated, respectively, using GraphPad Prism version 6 (GraphPad Software, Inc., San Diego, CA) with two-tailed t-tests. A *P*-value less than 0.05 was considered statistically significant. The heatmap of miRNAs/targets from the qRT-PCR data was constructed by R software (www.r-project.org). To investigate the biological significance of the DEGs that underwent AS in response to salt stress, those DEGs were subjected to GO analysis using the web software program WEGO [58] (http://wego.genomics.org.cn/).

## Results

### Different expression patterns of mRNAs and proteins in response to salt stress

**Fig. 1:**
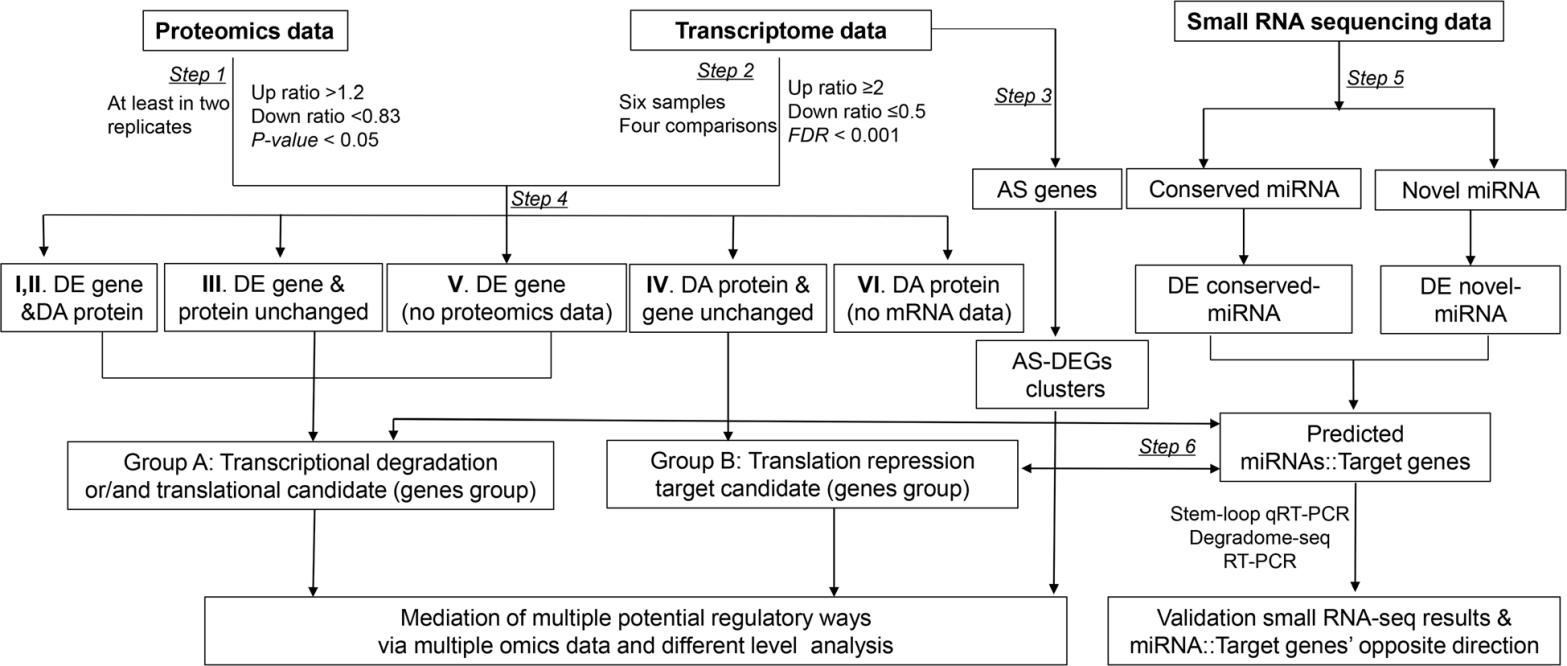
Schematic overview of proteomic and transcriptomic data generation and analysis workflow. Step 1: differential expression analysis of proteomic and Step 2: differential expression analysis of mRNA-seq data. Step 3: AS (alternative splicing) analysis using TopHat software and alternatively spliced differentially expressed genes (AS-DEGs) were obtained. Step 4: mapping of all proteins from the proteomic to mRNA-seq data and output of the two target groups. Among them, Group A contains translation repression and/or transcriptional degradation targets (contains differentially expressed genes and proteins whose levels were unchanged, both genes and proteins that were differentially expressed or no proteomic data). Group B contains potential targets of miRNA translational repression (differentially abundant proteins whose mRNA did not change or no mRNA data). Step 5: genome-wide analysis of differentially expressed conserved and novel miRNAs in both cotton genotypes under control and salt stress conditions. Step 6: psRNATarget analysis of both groups against miRNAs DE in the opposite direction combined with Group A and B data

**Fig. 2:**
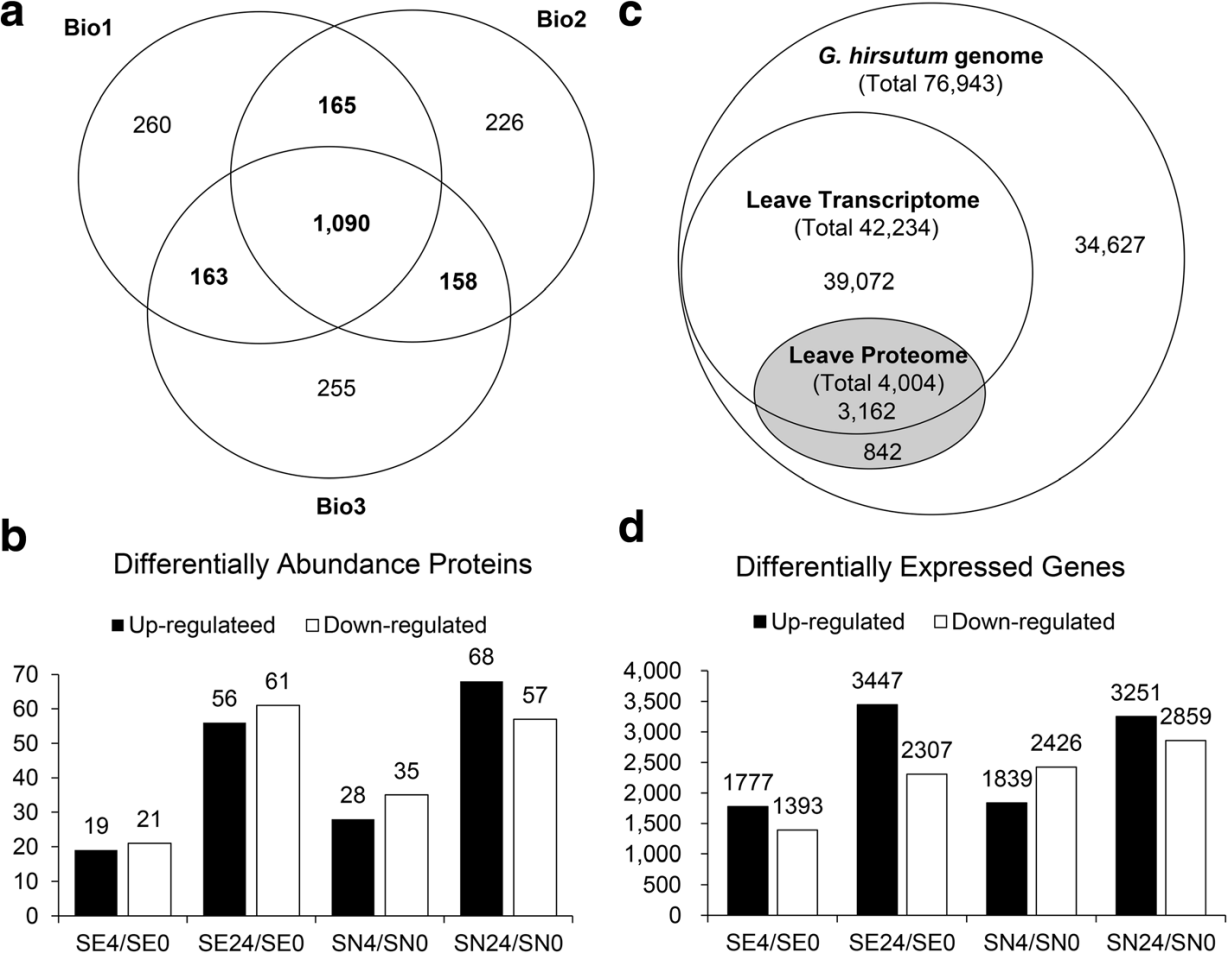
Multivariate data analysis overview. **a** Venn diagram showing the number of overlap and specific quantified proteins in three biological repeats. **b** Number of DAPs (differentially abundant proteins) at two time points after salt stress and in their respective control samples in the NH and E7 genotypes. **c** Congruency of the detected transcriptomic and proteomic data. **d** Number of DEGs (differentially expressed genes) at two time points after salt stress and in their respective control samples in the NH and E7 genotypes

A quantitative proteomic analysis of the six samples was performed using iTRAQ technology, and transcriptomic changes were monitored using the mRNA-seq and small RNA-seq. Figure 1 shows an overview of the data analysis strategy used in this study. The proteomic results when the *G. hirsutum* protein database was used, including peptide spectrum match quality (mass delta), number, distribution, protein sequence coverage and number per replicate, are presented in Additional file 2: Figure S1. Overall, a total of 4004 non-redundant proteins in the three biological replicates were identified (Additional file 3 a). A subset of 2810 proteins were identified using at least two peptides and in two replicates. Of these proteins, 2316 were quantified with iTRAQ ratios, and 1090 proteins were quantified in all three biological replicates (Fig. 2a, Additional file 3 b). Based on a 95% confidence level, 263 non-redundant proteins were differentially regulated under salt stress (Fig. 2b, Additional file 3 c-f).

To compare the abundance of these proteins and their change in abundance with respect to their respective gene transcript levels, a corresponding mRNA-seq experiment using the same samples was performed. A total of 42,234 expressed genes (transcripts) were detected in the leaves of both cotton genotypes in the presence and/or absence of salt stress after 4 and 24 h (Additional file 4 a). Subsequent analysis revealed that 3162 (78.97%, 3162/4004) of these genes were also identified from the proteomic analysis (Fig. 2c). In total, 9525 DEGs related to the salt response in the two cotton genotypes were detected (Fig. 2d, Additional file 4 a-d).

**Fig. 3:**
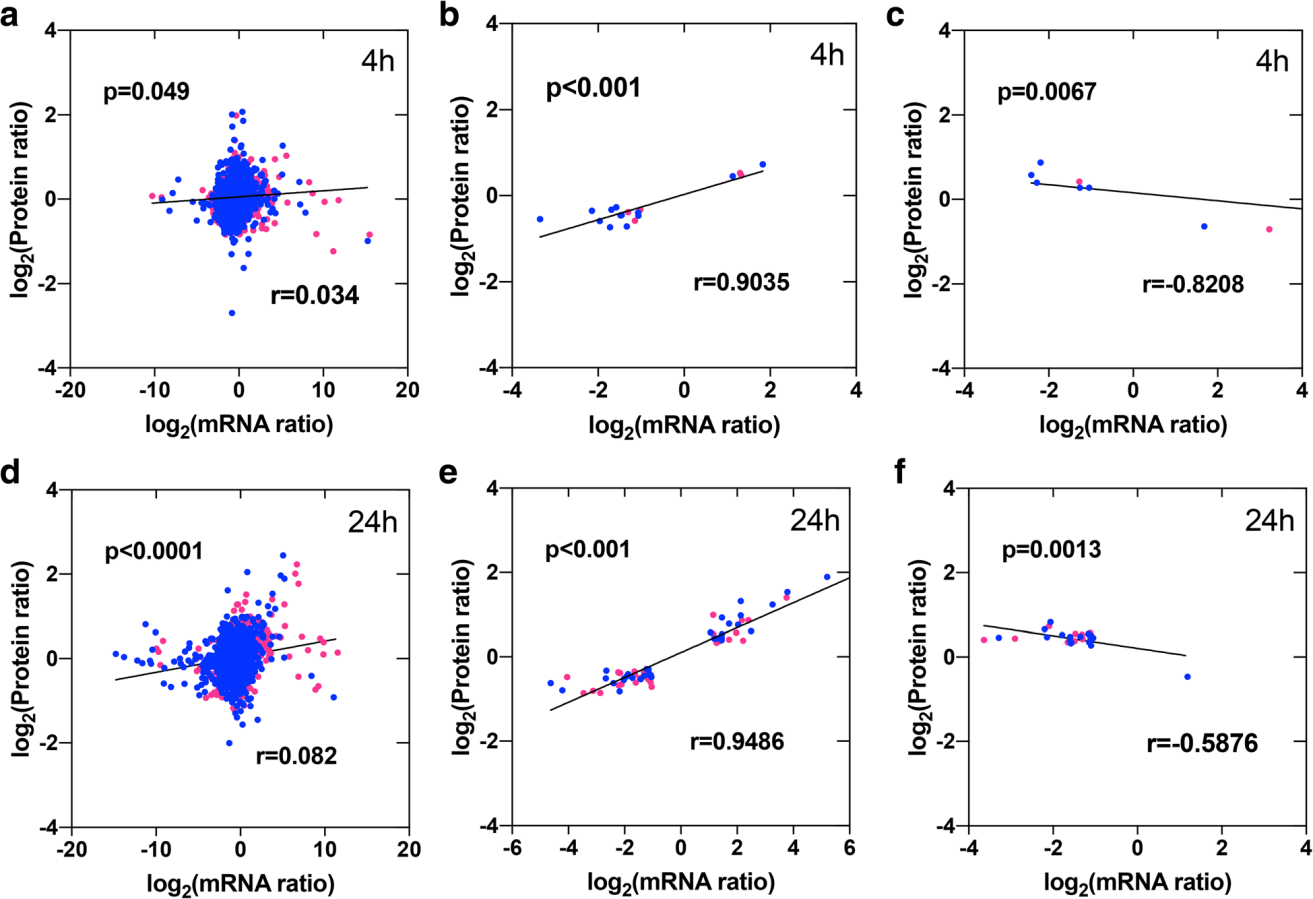
Concordance between changes in the abundance of genes and their encoded proteins. **a**–**c**, Scatterplots and correlations between protein and gene fold-changes for all gene/protein pairs after 4 h of salt treatment (**a**), the same direction of change for DAPs (differentially abundant proteins) and their associated DEGs (differentially expressed genes) (**b)**, and the opposite direction of change for DAPs and their associated DEGs (**c**). **d**–**f**, Scatterplots and correlations between protein and gene fold-changes for all gene/protein pairs after 24 h of salt treatment (**d)**, the same direction of change for DAPs and their associated DEGs (**e**), and the opposite direction of change for DAPs and their associated DEGs (**f**). The red plot indicates the E7 genotype, and the blue plot indicates the NH genotype

Quantitative global proteomic analysis was performed in parallel with the transcriptomic analysis, essentially revealing a different expression pattern observed at different levels. Globally, six distinct categories were identified based on the protein and gene expression profiles of SE4/SE0, SE24/SE0, SN4/SN0, and SN24/SN0 (Additional file 2: Figure S2 and Additional file 5). Correlations of all common genes/proteins between the proteomic and mRNA-seq results were analysed to investigate at which level protein accumulation is regulated (i.e., at the transcript level or post-transcript level or translational repression). The results showed that limited or no correlations were detected for all gene-protein pairs after 4 h (*r* = 0.034) and 24 h (*r* = 0.038) (Fig. 3a, d). However, stronger positive or negative correlations were observed between the DAPs and their corresponding mRNAs (both DAPs and DEGs in SE4/SE0, SE24/SE0, SN4/SN0, and SN24/SN0); these correlations were the same or were opposite the direction of change (*r* = 0.9035, − 0.8208 (4 h); 0.9486, − 0.5876 (24 h) (Fig. 3b, c, e and f).

### Biological pathway analysis based on mRNA and protein expression pattern responses to salt stress

#### Category I and II, DE at both the mRNA and protein levels

**Fig. 4:**
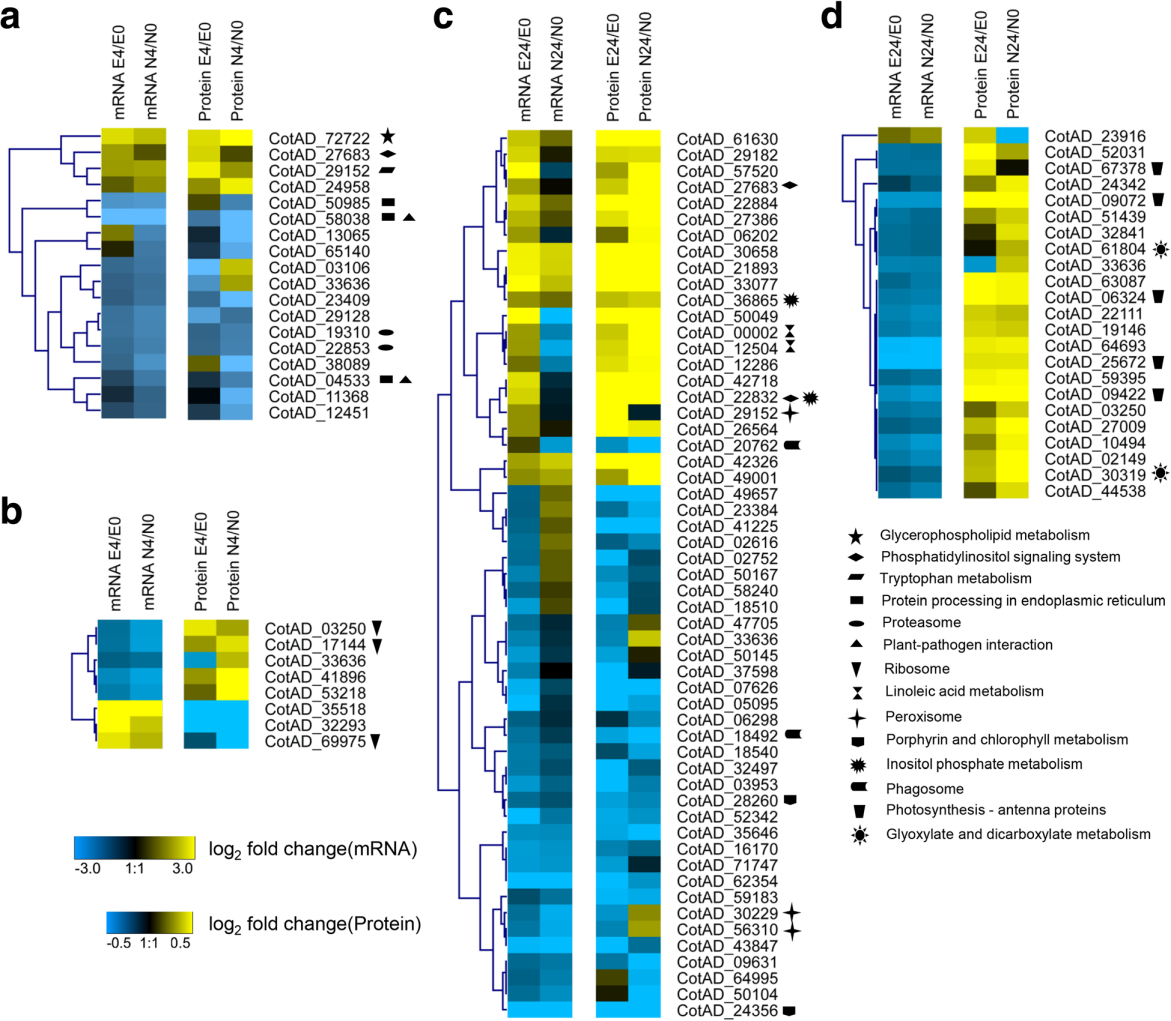
Hierarchical clustering analysis of mRNA-seq and proteomic data based on expression data. Heatmap of the proteins and mRNA expression ratios that have the same (**a**, **c**) or opposite (**b**, **d**) change tendencies after 4 h and 24 h of salt treatment, respectively. The expression profiles shown in the left and right panels are based on standardized log_2_ ratio values. The minimum and maximum displayed log_2_ ratios are ±3 for the transcriptomic data and ± 0.5 for the proteomics data. Black represents no significant change in expression. The KEGG pathway of genes/proteins showing significance are represented by various symbols behind the gene ID

In total, of 25 and 76 genes were significantly regulated at both the mRNA (≥ 2-fold difference and a FDR < 0.001) and protein (≥ 1.2-fold difference and *P*-value < 0.05) levels at 4 h and 24 h, respectively, under salt stress in the E7 and/or NH genotypes. Of these genes, 18 and 55 genes exhibited the same direction of change (category I), and 9 and 23 genes exhibited the opposite direction of change (category II) at the two levels (Fig. 4; Additional file 5 a, b).

To investigate the metabolic pathways responding to salt stress, each category was further investigated by KEGG pathway enrichment analysis (Additional file 5 c, d). The results showed that two (phosphatidylinositol signalling system and tryptophan metabolism) and three (protein processing in the endoplasmic reticulum, proteasome, and plant-pathogen interaction) pathways were differentially regulated at both the mRNA and protein levels in the E7 and NH genotypes, respectively, after 4 h of salt treatment (Fig. 4a). After 24 h, some KEGG pathways such as “phosphatidylinositol signalling system” and “peroxisome” completely differed in both DAPs and DEGs for same direction of change between E7 and NH (Fig. 4c). These results suggest that changes in gene expression cause corresponding changes at the protein level, and these genes and proteins associated with these processes highly differ between the two contrasting genotypes in response to salt stress. We also found that some proteins enriched in the “photosynthesis-antenna proteins” KEGG pathway exhibited opposite changes in direction between transcript and protein levels after 24 h of salt treatment (Fig. 4d). These differences indicated that proteins in the chloroplast have diverged from those of in the cytoplasm of eukaryotes or are controlled at the post-transcriptional level.

#### Category III and IV, DE at either the mRNA or protein level

The results of our proteomic and mRNA-seq analyses revealed that 502 (4 h) and 587 (24 h) genes were DE but that their corresponding proteins remained unchanged (category III). In addition, the 48 and 92 proteins at 4 h and 24 h were DE at the protein level, not at the mRNA level (category IV), under salt stress (Additional file 5 a, b).

Regarding category III genes/proteins at 4 h (only DE at the mRNA level), at the biological process level, the DEGs were especially involved in “glycolysis/gluconeogenesis, ether lipid metabolism, glycerophospholipid metabolism, fatty acid metabolism, and phenylpropanoid biosynthesis” in the E7 genotype as well as “ribosomes; carbon fixation in photosynthetic organisms; and stilbenoid, diarylheptanoid and gingerol biosynthesis”, which were in common in the NH genotype (Additional file 5 c). The DEGs whose proteins levels remained unchanged after 24 h were mainly involved in “photosynthesis”, “nitrogen metabolism”, “metabolic pathways” and “photosynthesis-antenna proteins” in both genotypes. The KEGG pathways of “porphyrin and chlorophyll metabolism” and “peroxisomes” were enriched only in the NH genotype (Additional file 5 d). Four significantly enriched KEGG pathways of category IV genes/proteins were identified (Additional file 5 d). For example, the “photosynthesis” and “pentose phosphate pathway” showed different expression at the protein level but not at the mRNA level. These results implied that these pathways were particularly affected at the post-transcriptional or post-translational level and that changes in mRNA (or protein) expression provided only limited insight into changes in protein (gene) expression between the two contrasting genotypes in response to salt stress.

### DEGs alternatively spliced in response to salt stress

**Fig. 5:**
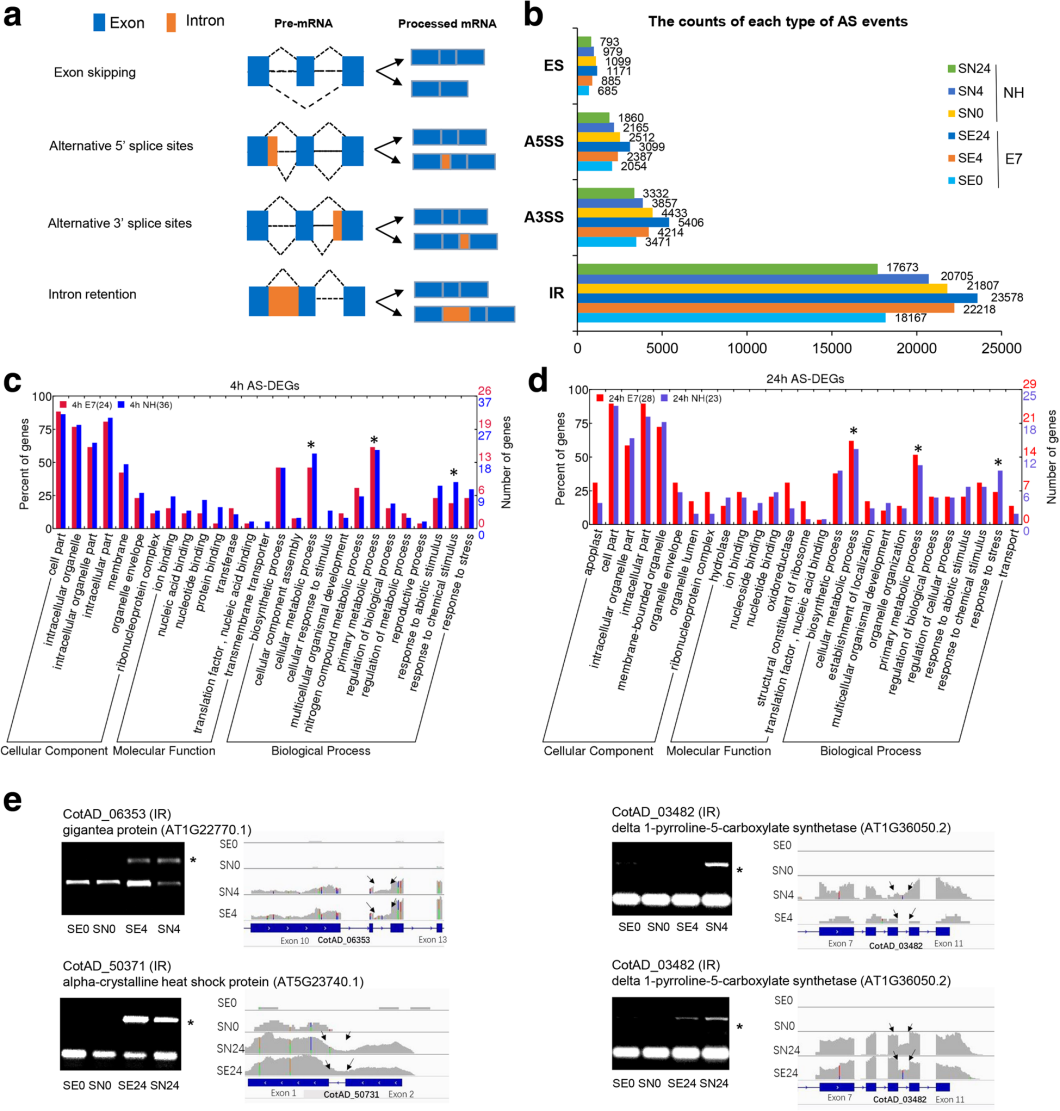
Genome-wide analysis of alternative splicing (AS) in both cotton genotypes under control and salt stress conditions. **a** The different types of AS, including IR, A5SS, A3SS and ES, are schematically illustrated. Exons are represented by boxes; introns, by lines. **b** The counts of each type of AS event in the control and after 4 h and 24 h of salt treatment of both cotton genotypes are shown. Comparison of functional categorization after 4 h (**c**) and 24 h (**d**) of salt stress revealed AS-DEGs between the E7 and NH genotypes. **e** Validation of the AS of selected genes detected by mRNA-seq via RT-PCR. The representative AS events (IR) in four stress-responsive genes were validated by RT-PCR and visualized using IGV browser. In the RT-PCR validation, the asterisk (*) on the right side denotes the AS form. In the IGV visualization, the exon–intron structure of each gene is given at the bottom of each panel. The grey peaks above the exon–intron structure indicate the RNA-seq read density across the gene, and the black arrows represent AS sites

Four major types of AS events and genes (intron retention (IR), exon skipping (ES), alternative 5′ splice sites (A5SS), and alternative 3′ splice sites (A3SS) were investigated (Fig. 5a). Clearly, more AS events occurred in the salt-treated E7 samples (SE4 and SE24) than in the control E7 samples (SE0). However, the opposite trend was observed for the sensitive genotype (NH) (Fig. 5b). These results indicate that the effect of overall AS events increased in the salt-sensitive genotypes as time progressed.

Among these AS events and involved genes, IR (more than 50%) was the most prevalent AS event under the salt stress and control conditions (Additional file 2: Figure S3a). There were 8945 (24.76%), 11,445 (30.94%), 11,535 (30.44%), 10,572 (28.43%), 9787 (26.87%), and 8626 (24.66%) genes associated with AS events identified from the mRNA-seq data (Additional file 2: Figure S3b). The Venn diagrams shown in Additional file 2: Figure S3c show the distribution of the common and specific AS genes among the four samples of each type (0, 4 h, and 24 h). After following the screening process shown in Additional file 2: Figure S3d, the AS-DEGs specifically induced by salt stress were identified by combining the DEG data of SE4/SE0, SE4/SE0, SE4/SE0, and SE4/SE0 (Additional file 6 a, b).

To determine which AS genes affected their corresponding protein abundance under salt stress conditions, we integrated the AS-DEG data sets with the four distinct categories of gene/protein data sets. Total amounts of 44 and 46 AS-DEGs, which were also quantified at the protein level after 4 h and 24 h of salt stress, respectively, in both genotypes, were identified. Not surprisingly, no significant differences were detected between the abundance of these proteins and their corresponding AS-DEGs (Additional file 6 c, d). These results indicate that, although the expression of some salt-responsive genes was affected by AS events, the abundance of their corresponding proteins remained unchanged under salt stress conditions. The GO-based functional annotation of genes after 4 h of salt stress revealed that the AS-DEGs in E7 encode enzymes involved in metabolic processes such as “primary metabolic process” (30.8% (E7); 24.3% (NH)), “cellular metabolic process” (46.2% (E7); 56.8% (NH)), and “nitrogen compound metabolic process” (30.8%; 24.3%); these percentages are greater than those detected in NH. In contrast, 30% of the proteins of AS-DEGs are involved in the “response to abiotic stimulus” in NH; this percentage is greater than that in E7 (Fig. 5c). Molecular function-based classification showed that the majority of the AS-DEGs after 24 h of stress were involved in “oxidoreductase activity” (31% (E7); 12% (NH)), “structural constituents of ribosomes” (17.2% (E7); 4% (NH)), “nucleotide binding” (13.8%; 8%), “ion binding” (24.1%; 20%), and “transferase activity” (10.3%; 8%) (Fig. 5d). Further, three genes with different AS patterns (IR) revealed by mRNA-seq after 4 h and/or 24 h of salt stress were selected and verified by RT-PCR (Fig. 5e). The results indicated that the subsets of genes responding to salt stress are spatiotemporally regulated both transcriptionally and post-transcriptionally in upland cotton. Ultimately, based on the absolute ratio of SE4 (or SE24)/SE0 more than that of SN4 (or SN24)/SN0, 26 genes that may be closely related to salt tolerance in upland cotton were identified (Additional file 6 e).

### Dynamic regulation of miRNAs in response to salt stress

The small RNA reads were mapped to the genome, and more than 68% of the unique reads and 80% of the total reads were mapped to the *G. hirsutum* genome (Additional file 7 a). In total, 59 known and 2930 novel miRNAs were identified (Additional file 7 b). To study the regulation of miRNAs in response to salt stress in cotton, a differential expression analysis of miRNAs between the control and salt-treated samples was performed. A total of 28 known miRNAs and 112 novel miRNAs were identified as DE in response to salt stress (Additional file 7 c). Especially, ghr-miR156a/b/d, ghr-miR160, ghr-miR166b, ghr-miR482b, ghr-miR2948-5p, ghr-miR2949a-5p, and ghr-miR7508 as well as 21 novel miRNAs were specifically down-regulated only in salt-tolerant genotype (E7) after 4 h and 24 h, and ghr-miR169a, ghr-miR394a/b, ghr-miR396a/b, ghr-miR482a, and ghr-miR7505 as well as 21 novel miRNAs were up-regulated, suggesting that these miRNAs might play more important roles in the cotton response to salt stress.

**Fig. 6:**
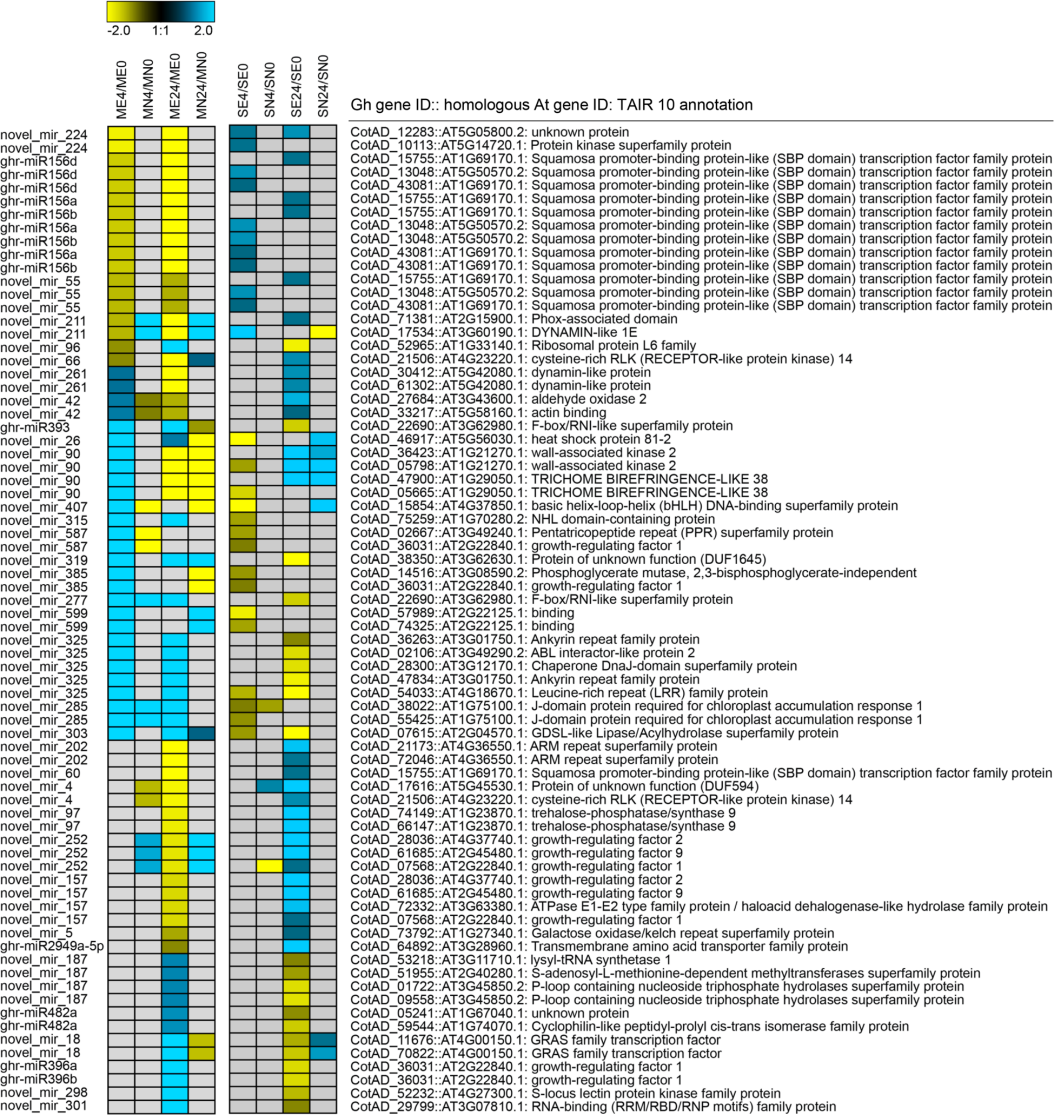
Combined view of expression levels between differentially expressed miRNAs (left panel) and their target differentially expressed genes (right panel) in the E7 and NH genotypes after 4 h and 24 h of salt stress

To characterize the possible functions of DE miRNAs in cotton, we first predicted their targets and further experimentally validated their expression levels. Using psRNATarget web software, the targets of the 28 known miRNAs and 106 novel miRNAs were determined (Additional file 2: Figure S4 and Additional file 7 d). Based on the negative correlations between the abundance of miRNAs and their targets and after the removal of only DE miRNAs in the NH genotype, eight known miRNAs and 30 novel miRNAs were detected and further analysed (Fig. 6). Four known miRNAs (ghr-miR156a/b/d and ghr-miR2949a-5p) and 11 novel miRNAs, all of which target plant development- and stress response-related proteins such as growth-regulating factors, SBP (squamosa promoter binding protein-like), protein kinases, and trehalose-phosphatase, were down-regulated. In addition, four known miRNAs (ghr-miR393, ghr-miR396a/b, and ghr-miR482a) and 15 novel miRNAs, all of which target growth-regulating factors, basic helix-loop-helix (bHLH) DNA-binding superfamily proteins, GRAS family transcription factors, and heat shock proteins, etc., were up-regulated after 4 h and/or 24 h of salt stress. In addition, four novel miRNAs (novel_mir_96, novel_mir_261, novel_mir_42, and novel_mir_90) exhibited opposite regulatory behaviour under salt stress between the two time points (Fig. 6). These results indicated that these miRNAs potentially regulated the expression of the salt tolerance-related genes in response to salt stress in cotton.

To determine which miRNAs both regulate gene expression and further induce changes in the abundance of their corresponding proteins under salt stress conditions, we integrated the DE miRNA-target gene sets with the four distinct categories of gene/protein data sets (Additional file 7 e). Predictions were carried out both for up-regulated miRNAs (ghr-miR394a, b) vs. down-regulated proteins and for four up-regulated miRNAs (ghr-miR482a, novel_mir_187, novel_mir_252, and novel_mir_385) vs. down-regulated genes after 4 or 24 h of salt exposure. The down-regulated proteins represented transketolase (chloroplast) and were enriched in “carbohydrate transport and metabolism”. The four down-regulated genes were enriched in the following categories: “post-translational modification and translation; translation; ribosomal structure and biogenesis; and carbohydrate transport and metabolism”. Another two novel DE miRNAs that positively correlated with DEGs (DEGs & protein ns) were targeted. Overall, only two and five genes/proteins were post-transcriptionally regulated by eight miRNAs after 4 h and 24 h of salt stress, respectively.

**Fig. 7:**
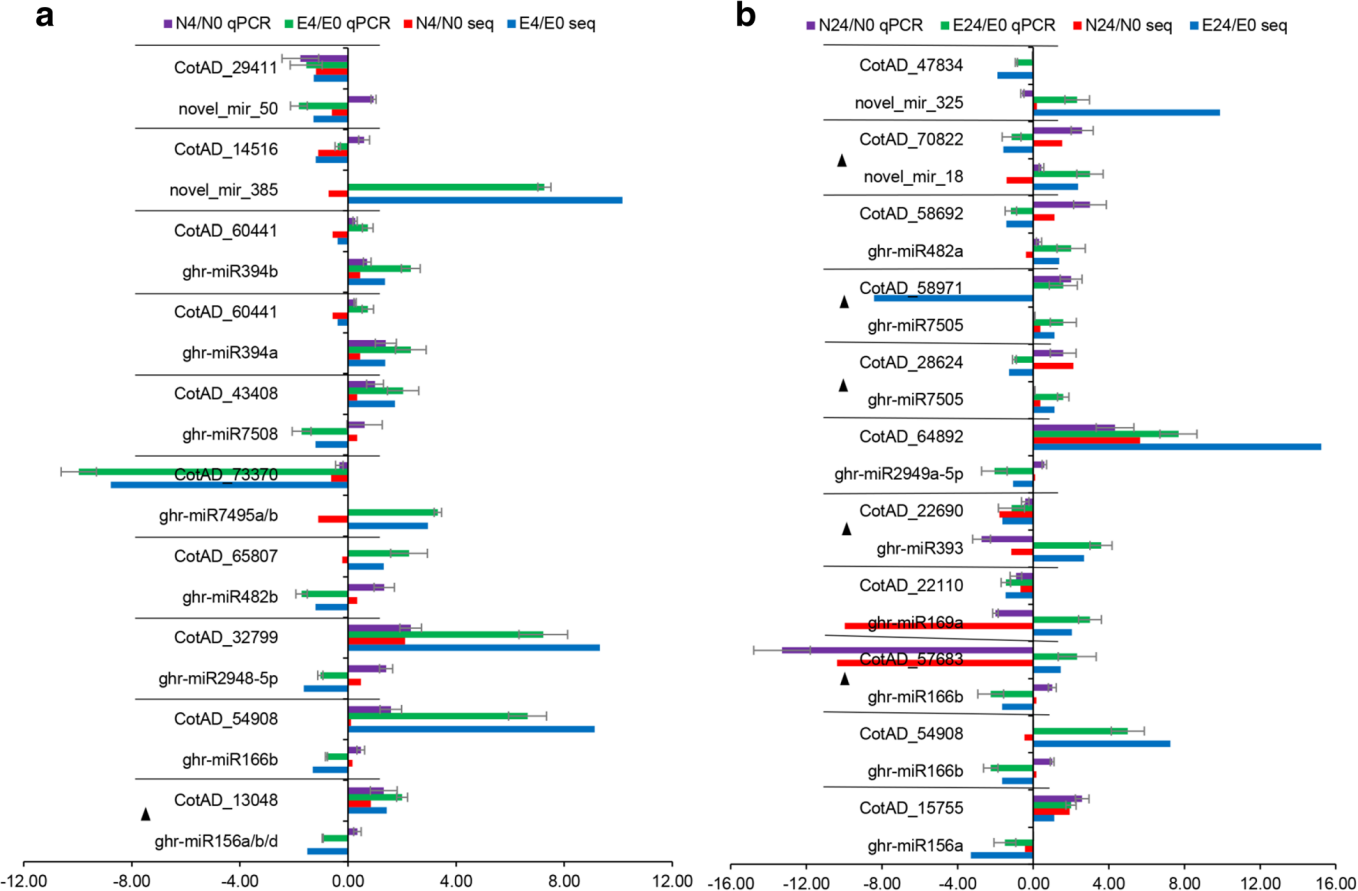
Validation of the expression patterns of twelve known and four novel differentially expressed miRNA-target modules by stem-loop qRT-PCR and qRT-PCR. qRT-PCR-based and stem-loop RT-PCR-based results showing a combined histogram of the inverse expression between miRNAs and their potential targets in E7 under salt stress for 4 h (**a)** and 24 h (**b**). miRNA expression (top) was validated by sequencing small RNAs isolated from control (ck) and salt-treated (4 h and 24 h) samples. The miRNA targets (bottom) were validated by mRNA-seq, and their expression levels were checked by qRT-PCR. Up- or down-regulation in expression was normalized to that of U6 snRNA or actin. The black triangle indicates that the conserved interactions of the sequenced miRNA targets were validated by the published degradome sequencing data using PAREsnip

To validate the miRNA cleavage targets, the degradome sequencing data from cotton seedlings and hypocotyls was used to discover the known and novel miRNA targets via the detection of cleaved miRNA targets [59]. Thirty-two and 153 miRNA-target pairs were further verified in the known and novel miRNA prediction results, respectively (Additional file 7 f). To validate the small RNA sequencing results, qPCR with 20 DE miRNAs was performed. The expression results of three samples from both genotypes in the presence and absence of salt treatment were similar to the results of the small RNA sequencing data (Additional file 2: Figure S5). To confirm the expression patterns of the salt-responsive miRNAs in cotton at the different time points, the expression patterns of 12 known and four novel DE miRNA-target modules were validated by qRT-PCR. Our results showed that the qRT-PCR and RT-PCR analyses of the majority of the DE miRNAs and their target genes displayed expression patterns similar to those revealed by the mRNA-seq data. Seven (4 h) and ten (24 h) miRNA-target pairs exhibited negative relationships at the expression level; these pairs included five that were validated by degradome sequencing (Fig. 7). These results indicated that post-transcriptional degradation of mRNA targets may be mediated by their corresponding miRNAs.

### Screening for distinct salt tolerance-related genes/proteins in tolerant and sensitive lines

To further identify the genes/proteins related to salt tolerance, we compared the results of the mRNA-seq and proteomic data analyses between the salt-tolerant and salt-sensitive genotypes. In accordance with the process shown in Additional file 2: Figure S6, we analysed 63 and 85 DEGs/DAPs between the E7 and NH genotypes after 4 and 24 h of salt stress, respectively, based on the stringent screening principles with respect to the mRNA-seq data (Additional file 2: Figure S2, categories I, II, III, and V) and proteomic data (Additional file 8 a, b). In the database, the *G. hirsutum* genes were also annotated with those of *A. thaliana* (TAIR 10); therefore, we obtained 1859 genes that were annotated with the GO term “response to osmotic stress” (including “response to salt stress”) and that were homologous to those of *A. thaliana* based on the GO annotation results (GO:0006970) in TAIR 10 (Additional file 8 c). We subsequently discovered 7 AS genes and 4 osmotic stress genes in 63 DEGs/DAPs in the 4 h data set as well as 6 AS genes and 11 osmotic stress genes in 85 DEGs/DAPs in the 24 h data set (Additional file 8 a, b). However, these genes/proteins were not miRNA targets. These results indicate that the identified salt tolerance genes/proteins are regulated only by AS, not by miRNAs. Without using the mRNA-seq data (Additional file 2: Figure S2; category VI), we also analysed the DAPs and identified 36 potential salt tolerance-related proteins (17 after 4 h of salt treatment and 27 after 24 h) (Additional file 8 d). To reveal the functional networks of these proteins, we performed a clustering analysis of the proteins based on well-established or predicted interactions from the STRING database (Additional file 8 e). The above combined protein interaction network (Additional file 2: Figure S7, Additional file 8 f) revealed that two main clusters were composed of ATP synthase (CotAD_74681) and cytochrome oxidase (CotAD_46197) in the mitochondria. Compared with those in NH, the majority of proteins in these clusters, which were involved in the response to stimuli, glucose catabolism and aerobic respiration, in E7 increased after 4 h and 24 h of salt stress. These results suggested that mitochondria, which are important organelles involved in energy metabolism, play an essential role in the synthesis of resistance proteins during the process of salt exposure.

Other proteins in other clusters were connected via interactions with ribosomal proteins, subtilase family proteins, rapid alkalization factors, zinc-finger proteins and transcription factors (Fig. 7). These proteins included cytochrome P450 (CotAD_18998), flavin-dependent monooxygenase (CotAD_20762), phosphatidyl inositol monophosphate 5 kinase (CotAD_40640), the phototropic-responsive NPH3 family protein (CotAD_66524) and an uncharacterized protein family (CotAD_52654), all of which may be key proteins involved in the response to salt stress.

## Discussion

### Regulation and association of various biological processes delineate salt tolerance signalling patterns in upland cotton

Herein, we first used an iTRAQ-based quantitative proteomic approach to profile proteins in six cotton samples of two contrasting cotton genotypes; these proteins represent two key stages of salt stress. We then compared the expression of those proteins with their mRNA levels and, using mRNA-seq and small RNA-seq approaches, identified several potential gene regulatory mechanisms. We found that transcriptional and post-transcriptional regulation might be related to the generation of AS and miRNAs. Several transcriptomic and proteomics studies involving the leaves, stems and roots of salt-treated cotton seedlings have been conducted prior to our work; these studies have accumulated a large body of information about the molecular mechanism of salt tolerance at both the RNA and protein levels [23–25, 27–31]. By exploiting the iTRAQ-based proteomic, transcriptomic, AS, and miRNA data of common samples, for the first time, we were able to analyse in depth the relationships among these important players in gene expression and reveal several mechanisms of gene regulation during salt stress.

**Fig. 8:**
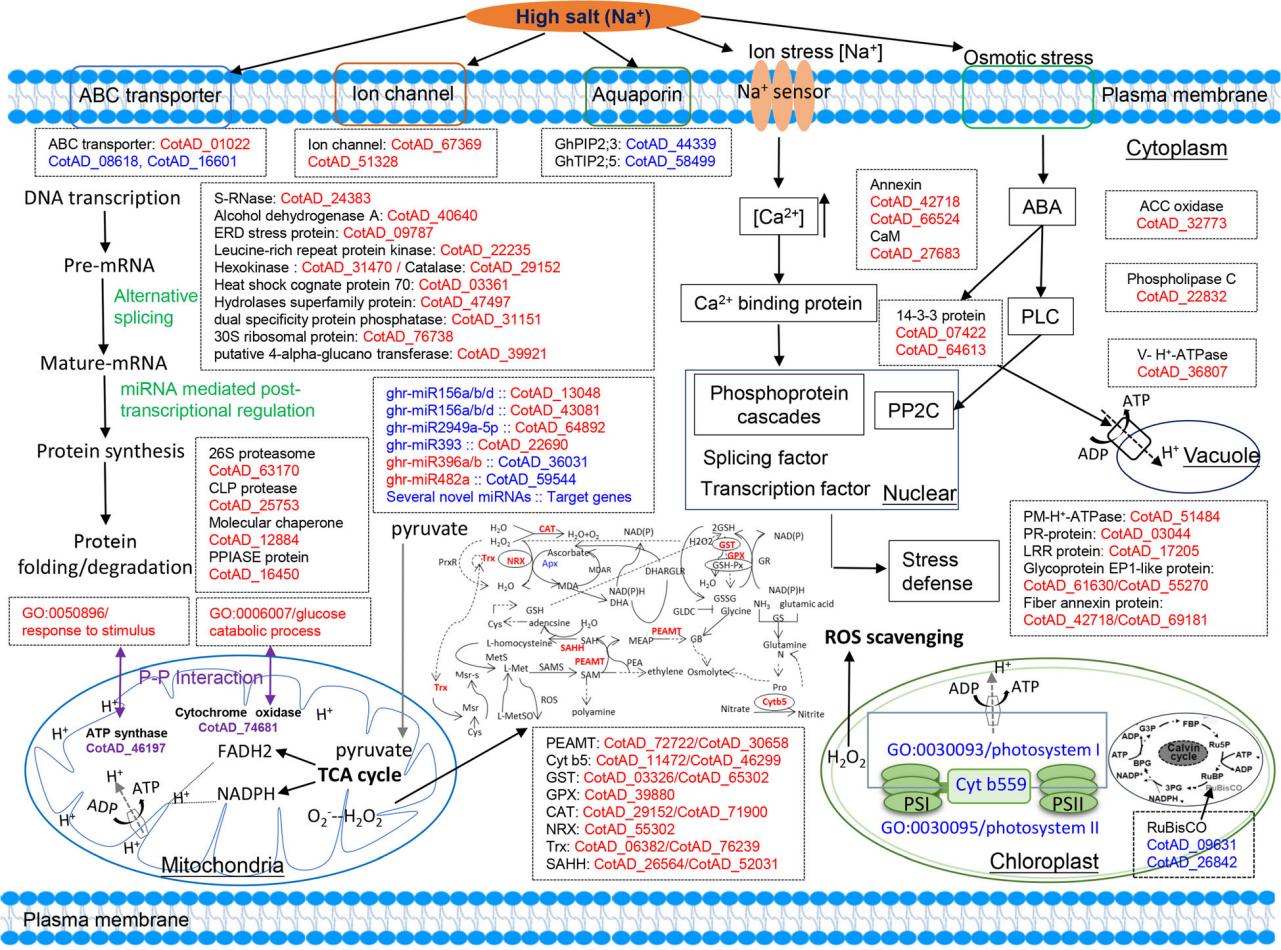
Schematic presentation of a plausible comprehensive salt-response and defence model in salt-tolerant genotypes. Most key DAPs (differentially abundant proteins) and DEGs (differentially expressed genes) as well as differentially expressed miRNAs are integrated with dotted boxes and indicated in red (up-regulated after 4 h and/or 24 h of NaCl treatment relative to their expression in control samples) or blue (down-regulated), respectively. In the mitochondria, the two core proteins, ATP synthase (CotAD_74681) and cytochrome oxidase (CotAD_46197), are associated with interactions with proteins involved in response to stimuli. Glucose catabolic processes are represented by purple font. All information concerning these proteins, genes and miRNAs that were differentially expressed under salt stress can be found in Additional files 5, 6 and 7

In addition to comprehensively analysing the global changes in mRNA, miRNA, and protein profiles, a hypothetical schematic for systematic salt tolerance signalling patterns was put forth in this study (Fig. 8). The combination of the proteomic and mRNA-seq data revealed strong overall agreement between changes in both mRNA and protein levels for both induced and repressed proteins/genes in response to salt stress. We identified several biological processes, such as the “phosphatidylinositol signalling system”, “tryptophan metabolism”, “inositol phosphate metabolism”, and “glyoxylate and dicarboxylate metabolism”, that may be less affected by the extra regulatory mechanisms of salt tolerance. However, for the majority of the strongly DAPs, no significant change in their corresponding mRNA levels was observed. Similarly, for the majority of the DEGs, no significant change in their corresponding protein levels was observed. These results might have occurred because of post-transcriptional/translational regulation of protein activity to avoid a de novo cycle of synthesis after stress [60, 61]. On the other hand, some proteins may not be urgently needed under stress conditions and must be synthesized at the mRNA level to be translated in a short time to perform their function [62].

The results showed that regulation and association delineated various important biological processes involved in salt tolerance signalling patterns in upland cotton. When plants experience sudden salt stress, salt perception is detected by ion and osmotic stress signals. Signalling molecules such as calcium, redox modulators, phytohormones (such as abscisic acid (ABA) and ethylene (ET)), and protein kinases, all of which play independent roles via different pathways, were identified in our data set (Fig. 8, Additional file 5). These signals are first sensed by receptors on membranes; many membrane transport pathways, including ion channels and ion carriers such as ion channel proteins (CotAD_67369 and CotAD_67369) and ABC transporters (CotAD_01022, CotAD_08618, and CotAD_16601), also participate in this signal transduction. We also identified two annexin proteins (CotAD_42718 and CotAD_66524) that were significantly up-regulated at the gene and protein levels after 4 and/or 24 h of salt treatment in the E7 genotype. The *GhAnn1* gene in cotton has been cloned; this gene plays an important role in the abiotic stress response, and overexpression of *GhAnn1* in transgenic cotton improves salt and drought tolerance [63]. Expressed in most eukaryotic cells, 14–3-3 proteins are general regulatory factors. In our study, two 14–3-3 proteins (CotAD_07424 and CotAD_64613) were up-regulated after 24 h of salt stress. It was recently reported that 14–3-3 proteins could inhibit the salt tolerance salt overly sensitive (SOS) pathway by interacting with SOS2 and repressing its kinase activity [64]. Thus, an opportunity will exist to understand the effects of different salt tolerance mechanisms apart from the SOS pathway. Some salt-responsive downstream genes, especially those coding for pathogenesis-related (PR) (CotAD_03044) and leucine-rich repeat (LRR) family proteins (CotAD_17205), ERD stress proteins (CotAD_09787) and fibre annexin proteins (CotAD_42718 and CotAD_69181), can also provide good clues for understanding the mechanism responsible for salt tolerance.

As another aspect of salt tolerance, the ROS scavenging system can play an effective regulatory role in protecting membranes and macromolecules. In our study, several genes/proteins related to detoxification were up-regulated at the different time points under salt stress conditions (Fig. 8, Additional file 5). Here, are three major approaches to the redox scavenging system. First, H_2_O_2_ can be reduced to H_2_O in the catalase (CAT) pathway, which is mainly localized in the peroxisome. mRNA-seq studies have shown that the expression of CAT genes (CotAD_29152 and CotAD_71900) increases. Second, the peroxiredoxin/thioredoxin (PrxR/Trx) pathway is a central antioxidant defence system in plants and constitutes a multigenic family whose members are involved in ROS metabolism. Trx (CotAD_06382 and CotAD_76239), a key protein in this pathway, is also affected by salinity in other species [65, 66]. Third, the glutathione peroxidase (GPX) pathway is generally considered a major enzymatic defence system against oxidative membrane damage. Glutathione S-transferases (GSTs) have GPX activity and can use glutathione (GSH) to reduce organic hydroperoxides of fatty acids [67]. In our study, GPX (CotAD_39880) and GST (CotAD_03326 and CotAD_65302) genes/proteins increased in response to salt stress. In fact, most GSTs increase in species such as Arabidopsis [68], rice [69] and bread wheat when under salt stress conditions [20].

In general, glycine betaine (GB) is an important osmoprotectant for maintaining osmotic pressure and conferring tolerance to salinity, drought, and other stresses [70, 71]. GB is synthesized via the two-step oxidation of choline (Cho) in the chloroplast. Therefore, the Cho supply determines how much GB is synthesized. In the Cho and GB biosynthesis pathways in plants, the rate-limiting enzyme of the three-step methylation reaction is phosphoethanolamine N-methyltransferase (PEAMT; EC 2.1.1.103) [72]. In our study, one homologous pair – that of the *GhPEAMT* gene (CotAD_72722 and CotAD_30658) – exhibited up-regulated (4~ 64-fold in E7) mRNA and protein levels after 4 h and 24 h of salt treatment (Additional file 5). Cho biosynthesis in the Arabidopsis *peamt* mutant t365 is reduced by approximately 64%, resulting in mutant plants that are highly sensitive to salinity; this finding confirms that the PEAMT gene can promote plant resistance to salt [73]. Furthermore, overexpression of the PEAMT gene in tobacco increases the level of phosphocholine by 5-fold and that of Cho by 50-fold and leads to approximately 30-fold greater levels of synthesized betaine, without affecting plant growth. This finding indicated that the PEAMT gene significantly increased the Cho content in the transgenic plants and alleviated the lack of endogenous Cho content during the synthesis of betaine [72]. These findings suggest that we can further improve the ability of cotton cells to synthesize GB by introducing a synthesis pathway that enhances Cho, conferring improved salt tolerance to transgenic plants.

According to the *A. thaliana* STRING database, we predicated an interaction network of 158 genes/proteins, which revealed two main clusters composed of ATP synthase (CotAD_74681) and cytochrome oxidase (CotAD_46197) in the mitochondria (Fig. 8, Additional file 8 f). As such, mitochondria, as important organelles involved in energy metabolism, play essential roles in the synthesis of resistance proteins during the process of salt exposure. Mitochondrial function is one of the major cellular processes involved in responses to salt stress in plants. These responses include alterations to the activity of the tricarboxylic acid cycle, which can influence the carbon balance between growth, the supply of reductants to the electron transport chain, and the supply of ATP and reductants provided by the mitochondria to drive essential reactions in the rest of the cell to avoid salt toxicity [74]. In addition, several subunits of ATP synthase exhibit increased abundance in plants tolerant to salt stress under salt stress conditions, while some sensitive plants exhibit decreased relative abundance of ATP synthase subunits. Although the two genes (CotAD_74681 and CotAD_46197) in the mitochondria were not identified in our study, the important proteins with which they interact in the cytoplasm may be associated with salt tolerance. This possibility could be indirectly supported by the relationship between salt stress signalling pathways and mitochondrial metabolic processes in C3 plants in response to salt induction [75].

### Alternative splicing acts as a regulatory mechanism linked to salt tolerance

In plants, the AS of precursor mRNA (pre-mRNA) is an important gene regulatory process that potentially regulates physiological processes at different development stages or environmental conditions such as salt stress [76, 77]. In this study, we systematically analysed the transcriptome of salt-treated samples; the results revealed that more than 50% of the intron-containing genes are alternatively spliced (Additional file 2: Figure S3a). This number is higher than that in Arabidopsis and rice, but IR, which was the most abundant AS event identified in this study, still remains a typical feature in plants [78, 79]. Interestingly, the number of AS genes in the salt-tolerant genotype gradually increased as the duration of salt stress increased, but the opposite trend was observed for the salt-sensitive genotype (Fig. 5a). These marked AS events under salt stress conditions might play a functional role in regulating the response and tolerance of upland cotton to stress.

In this study, we first obtained the AS-DEG information in response salt stress by the screening in accordance with the method shown in Additional file 2: Figure S3c, and then combined that information with the proteomics data. In total, 44 and 46 AS-DEGs in which the AS event affected the corresponding protein abundance under salt stress conditions in both genotypes after 4 h and 24 h, respectively, were identified (Additional file 6 c, d). These results suggest that these salt-responsive genes were affected by AS at the post-transcriptional regulatory level. Furthermore, the results of the GO analysis revealed that more than 60% of the AS-DEGs code for enzymes involved in metabolic processes, and another 30% of AS-DEGs code for proteins involved in the response to chemical stimuli (Fig. 5d, Additional file 6 e). In fact, most of the AS events reported in response to salt stress concern genes with regulatory roles, covering all levels of regulation of gene expression [80–85]. As is known, as the duration of salt stress increases, a large number of stress-inducible pre-mRNAs is produced; under these conditions, the cells would need to immediately recruit a large number of splicing factors and other factors for co-transcription or post-transcriptional regulation. AS-based regulation of genes might greatly enhance and amplify the signal transduction cascade in response to stress, affecting such components as transcription factors [80, 85, 86], ROS [81, 87], RNA-binding proteins [88]. We found that these genes mainly compose functional categories, e.g., “nucleic acid binding”, “oxidoreductase activity”, “peroxidase activity” and “ion binding” (Additional file 6 e). Of course, determining whether downstream genes are regulated by upstream genes with an AS pattern requires additional study. However, these results prove that the role of splicing regulation may be different from that of other regulatory genes with respect to further amplification of the signal cascade or direct effects on downstream genes.

This discussion has referred only to global changes in AS under salt stress conditions and the possible mechanisms involved in the regulation of different genes expressed at variable levels while non-significantly different changes in protein levels simultaneously occur. In practice, AS plays an important role in regulating the response and tolerance of upland cotton.

### miRNA expression can affect the salt sensitivity of cotton genotypes, but not directly reflected in protein abundance level

There has recently been increasingly evidence suggesting that miRNAs, as post-transcriptional gene regulators, play a critical role in abiotic and biotic stress responses [35]. To date, efforts have been made to study salinity stress-regulated miRNAs in upland cotton [34, 89, 90].

In this study, we identified 59 known and 2930 novel miRNAs (Additional file 7 b). Among the miRNAs, 28 known miRNAs and 112 novel miRNAs were identified to be DE in response to salt stress. Interestingly, the majority of the responding miRNAs were either differentially regulated only in the salt-tolerant genotype or exhibited distinct expression trends (Additional file 7 c). Compared with the results of known miRNA expression profiles from previous studies, the results showed that miR156, miR166, miR169, miR393, miR396, and miR482 were DE only in the salt-tolerant genotype (E7) but not significantly in the salt-sensitive genotype (NH) or in TM-1 genotype [90]. These results might suggest that these conserved miRNAs are not equally affected by the different genotypes involved in the regulation of gene expression. In addition, ghr-miR7495a/b, ghr-miR7505, ghr-miR7508, ghr-miR827a/b/c, ghr-miR2948-5p, and ghr-miR2949a-5p were first identified to respond to salt stress after 4 h or 24 h in our study. On the other hand, their predicted targets revealed a variety of biological processes, including transcription factors, the cell cycle, phyllome development, metabolic processes, responses to osmotic stress, signal transduction, and lipid transport (Fig. 6). For example, based on the predicted targets, ghr-miR156a/b/d may regulate 32 SBP transcription factors and signal transduction-related genes, which have been validated to be miR156 targets and are involved in floral development in Arabidopsis [91] and rice [92]. In addition, ghr-miR396a/b was predicted to target growth-regulating factors. Similar results were also reported in previous studies [34, 89]. We identified three novel miRNAs (novel_mir_385, novel_mir_587, and novel_mir_530) whose targets were also growth-regulating factors; these miRNAs were validated by degradome sequencing (Additional file 7 d, f). Overall, these similarly regulated miRNAs may represent the fundamental mechanism of adapting to salt stress, and the differentially regulated miRNAs might explain the distinct salt sensitivities between the two cotton genotypes.

In plants, miRNAs are known to post-transcriptionally repress genes by guiding the ARGONAUTE-mediated cleavage of target mRNAs or by translational repression [93]. In this study, small RNA-seq samples were also subjected to iTRAQ-based comparative proteomic analysis to determine whether miRNAs regulate gene expression and further induce changes in the abundance of their corresponding proteins under salt stress conditions (Additional file 7 e). Unfortunately, only two (known miRNAs targets) and five (novel miRNAs targets) genes/proteins were detected to be post-transcriptionally regulated by eight miRNAs after 4 h and 24 h of salt stress, respectively. In actuality, we did not expect all of the identified genes to be predicted targets of miRNAs for certain reasons. First, the differential expression of mRNA and protein occurs because miRNAs are not involved in certain processes or with certain components, such as mRNA splicing [94] and cis-siRNAs [95]. Second, the design of the psRNATarget algorithm, which is considered one of the most rigorous methods, may limit the number of computer predictions. The algorithm considers only the coding region (CDS) and excludes the target sites within the 3′-untranslated region (UTR). Therefore, we cannot exclude that spatial differences exist between miRNA expression patterns at the protein level in response to salt stress.

## Conclusion

We comprehensively analysed the global changes in mRNA, miRNA, and protein profiles in response to salt stress in two contrasting salt-tolerant cotton genotypes. Overall, 2316 proteins were quantified with iTRAQ ratios, and 1090 proteins were quantified in all three biological replicates. A total of 42,234 expressed genes were detected in the leaves of both cotton genotypes in the presence and/or absence of salt stress after 4 and 24 h. The results of additional analyses revealed that 3162 genes/proteins (78.97%) of the 4004 proteins of the proteome were detected. The results of the association analysis between the proteomic and mRNA-seq data showed that some genes were DE both at the proteomic and mRNA levels, but we also found that the majority of the strongly DAPs exhibited no significant change in their corresponding mRNA levels. We then provided evidence that several salt stress-responsive proteins can alter miRNAs and modulate AS events in upland cotton. The results of the stringent screening of the transcriptome and proteome of both the salt-tolerant and salt-sensitive genotypes further identified 63 and 85 candidate genes/proteins related to salt tolerance after 4 and 24 h of salt stress, respectively. Finally, we predicted an interaction network comprising 158 genes/proteins and then discovered two main clusters composed of ATP synthase (CotAD_74681) and cytochrome oxidase (CotAD_46197) in the mitochondria. Overall, we provided a possible schematic for the systematic salt tolerance model; this schematic reveals multiple levels of gene regulation in cotton in response to salt stress.

## Additional files

Additional file 1: **Table S6.** Primers for miRNA and target genes for qRT-PCR and stem-loop RT-PCR. (XLSX 16 kb)

Additional file 2: **Figure S1.** Basic information of protein identification. a) Mass delta; b) Peptide number; c) Protein mass; d) Coverage; e) Basic identity; f) Repeatability. **Figure S2.** Integrative analysis of the proteomic and mRNA-seq data. **Figure S3.** Global comparison of AS events and genes. a) The number and percentage of IR, A5SS, A3SS and ES events under the control and salt stress conditions in both genotypes. b) The number and percentage of AS genes and non-AS genes. c) The number of specific and common AS genes after 4 h and 24 h of salt treatment. d) The combination of all identified specific and common data sets of AS genes after 4 h and 24 h of salt treatment, with corresponding DEG data. **Figure S4.** The number of DE identified known and novel miRNAs in four comparisons. The DE identified known and novel miRNA targets were predicted using psRNATarget web software (http://plantgrn.noble.org/psRNATarget/). **Figure S5.** RT-PCR assay-based (left panel) and small RNA-seq-based (right panel) expression profiles of 12 known and 5 novel miRNAs from the control (0 h) and salt-treated (4 and 24 h) seedlings of both cotton genotypes. **Figure S6.** Flowchart analysis of the distinct genes/proteins between E7 and NH under salt stress conditions. **Figure S7**. PPI network of the combination of genes and proteins that display contrasting expression patterns in the E7 and NH genotypes. The PPI interactions with a combined score greater than 160 in the STRING database were extracted to construct the network with Cytoscape version 3.5.0 (http://www.cytoscape.org/). Orange boll represent DE transcripts or proteins, and blue coloring indicates the interactions of their target proteins;The red and blue triangle represent the AS-DEGs in the E7 genotype after 4 h and 24 h of salt treatment, respectively. (PDF 968 kb)

Additional file 3: **Table S1.** Summary of iTRAQ-based proteomic data. (XLSX 3294 kb)

Additional file 4: **Table S2.** Summary of mRNA-seq data. (XLSX 2064 kb)

Additional file 5: **Table S3**. Summary of four types of proteomic and mRNA-seq data. (XLSX 1082 kb)

Additional file 6: **Table S4.** Summary of AS-DEGs and their corresponding protein information after 4 h and 24 h of salt stress. (XLSX 505 kb)

Additional file 7: **Table S5.** Summary of small RNA-seq data. (XLSX 774 kb)

Additional file 8: **Table S7.** List of the 158 distinct genes/proteins between E7 and NH under salt stress conditions, as well as PPI information. (XLSX 27938 kb)

## Abbreviations

AS-DEG: Alternatively spliced differentially expressed gene
DAP: Differentially abundant protein
DEG: Differentially expressed gene
E7: Earlistaple 7
FDR: False discovery rate
iTRAQ: Isobaric tag for relative and absolute quantitation
KEGG: Kyoto Encyclopedia of Genes and Genomes
MGF: Mascot generic format
NH: Nan Dan Ba Di Da Hua
PPI: Protein–protein interaction
qRT-PCR: Quantitative reverse transcription-PCR
SCX: Strong cation exchange
