# Supplementary material for: Integration of proteomic and transcriptomic profiles reveals multiple levels of genetic regulation of salt tolerance in cotton
Source: BMC Plant Biol. 2018 Jun 20;18:128. doi: 10.1186/s12870-018-1350-1 (PMC6011603; doi:10.1186/s12870-018-1350-1)
Supplement: Supplementary file 2 — Figure S1. Basic information of protein identification. a) Mass delta; b) Peptide number; c) Protein mass; d) Coverage; e) Basic identity; f) Repeatability. Figure S2. Integrative analysis of the proteomic and mRNA-seq data. Figure S3. Global comparison of AS events and genes. a) The number and percentage of IR, A5SS, A3SS and ES events under the control and salt stress conditions in both genotypes. b) The number and percentage of AS genes and non-AS genes. c) The number of specific and common AS genes after 4 h and 24 h of salt treatment. d) The combination of all identified specific and common data sets of AS genes after 4 h and 24 h of salt treatment, with corresponding DEG data. Figure S4. The number of DE identified known and novel miRNAs in four comparisons. The DE identified known and novel miRNA targets were predicted using psRNATarget web software (http://plantgrn.noble.org/psRNATarget/). Figure S5. RT-PCR assay-based (left panel) and small RNA-seq-based (right panel) expression profiles of 12 known and 5 novel miRNAs from the control (0 h) and salt-treated (4 and 24 h) seedlings of both cotton genotypes. Figure S6. Flowchart analysis of the distinct genes/proteins between E7 and NH under salt stress conditions. Figure S7. PPI network of the combination of genes and proteins that display contrasting expression patterns in the E7 and NH genotypes. The PPI interactions with a combined score greater than 160 in the STRING database were extracted to construct the network with Cytoscape version 3.5.0 (http://www.cytoscape.org/). Orange boll represent DE transcripts or proteins, and blue coloring indicates the interactions of their target proteins;The red and blue triangle represent the AS-DEGs in the E7 genotype after 4 h and 24 h of salt treatment, respectively. (PDF 968 kb) [file 12870_2018_1350_MOESM2_ESM.pdf]

Supplementary Materials

Integration of proteomic and transcriptomic profiles reveals multiple levels of genetic regulation of salt tolerance in cotton

Zhen Peng, Shoupu He, Wenfang Gong, Feifei Xu, Zhaoe Pan, Yinhua Jia, Xiaoli Geng, Xiongming Du*

* To whom correspondence should be addressed

Address: State Key Laboratory of Cotton Biology, Institute of Cotton Research, Chinese Academy of Agricultural Sciences, Anyang, Henan 455000, China

Telephone: +086-0372-2562252,

Fax: +086-0372-2562256

Email: dujeffrey8848@hotmail.com

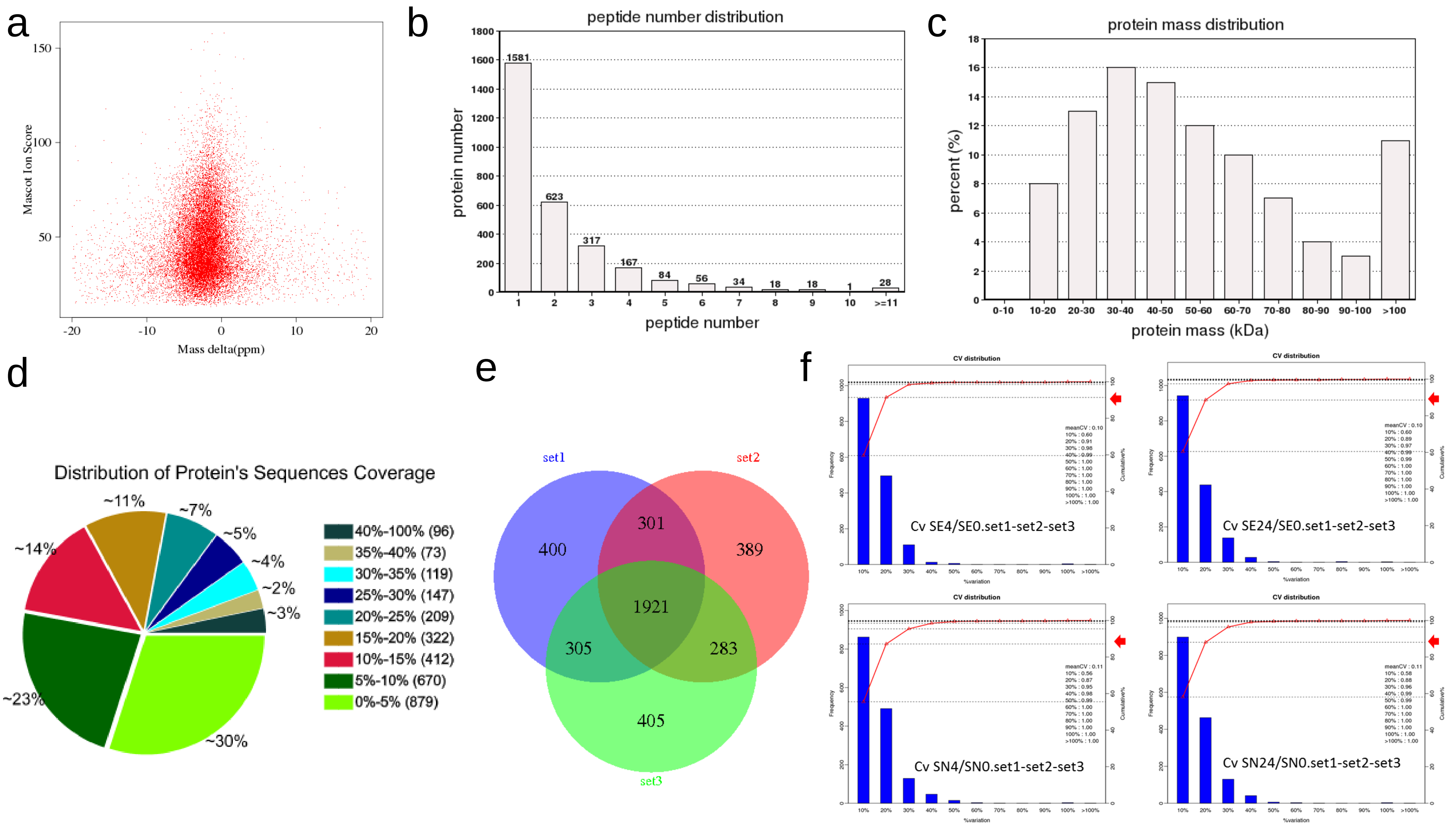

Figure S1: Basic information of protein identification. (a) Mass delta; (b) Peptide Number; (c) Protein Mass; (d) Coverage; (e) Basic identify; (f) Repeatability.

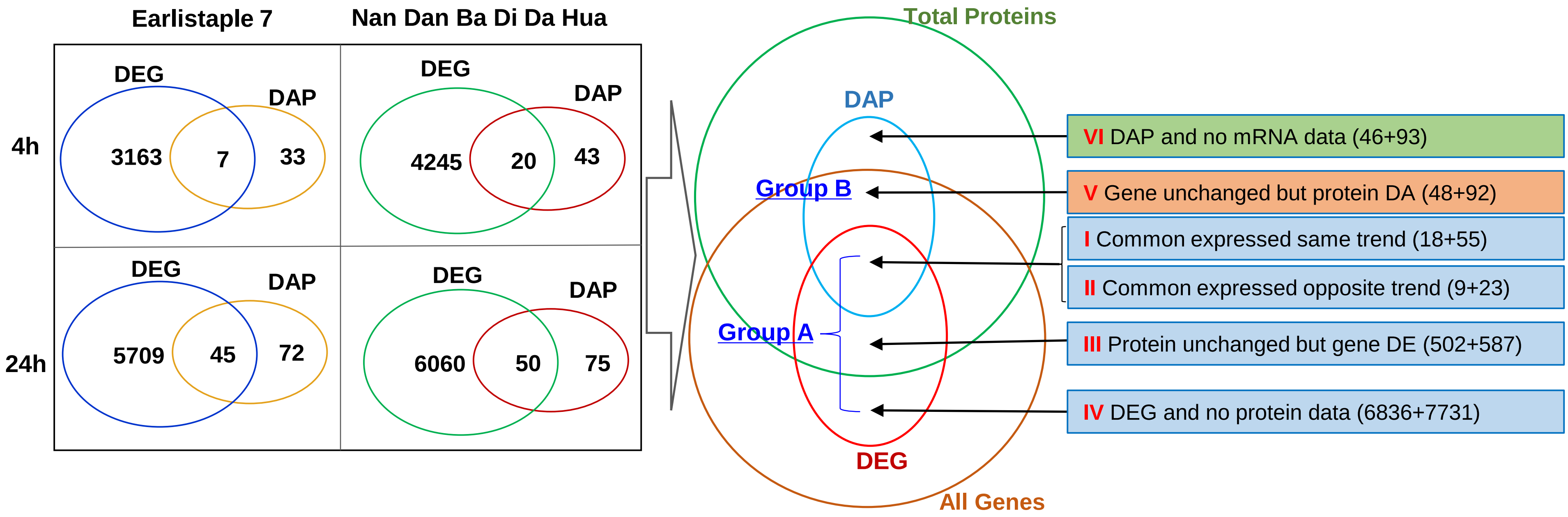

Figure S2： Integrative analysis of the proteome and mRNA-seq

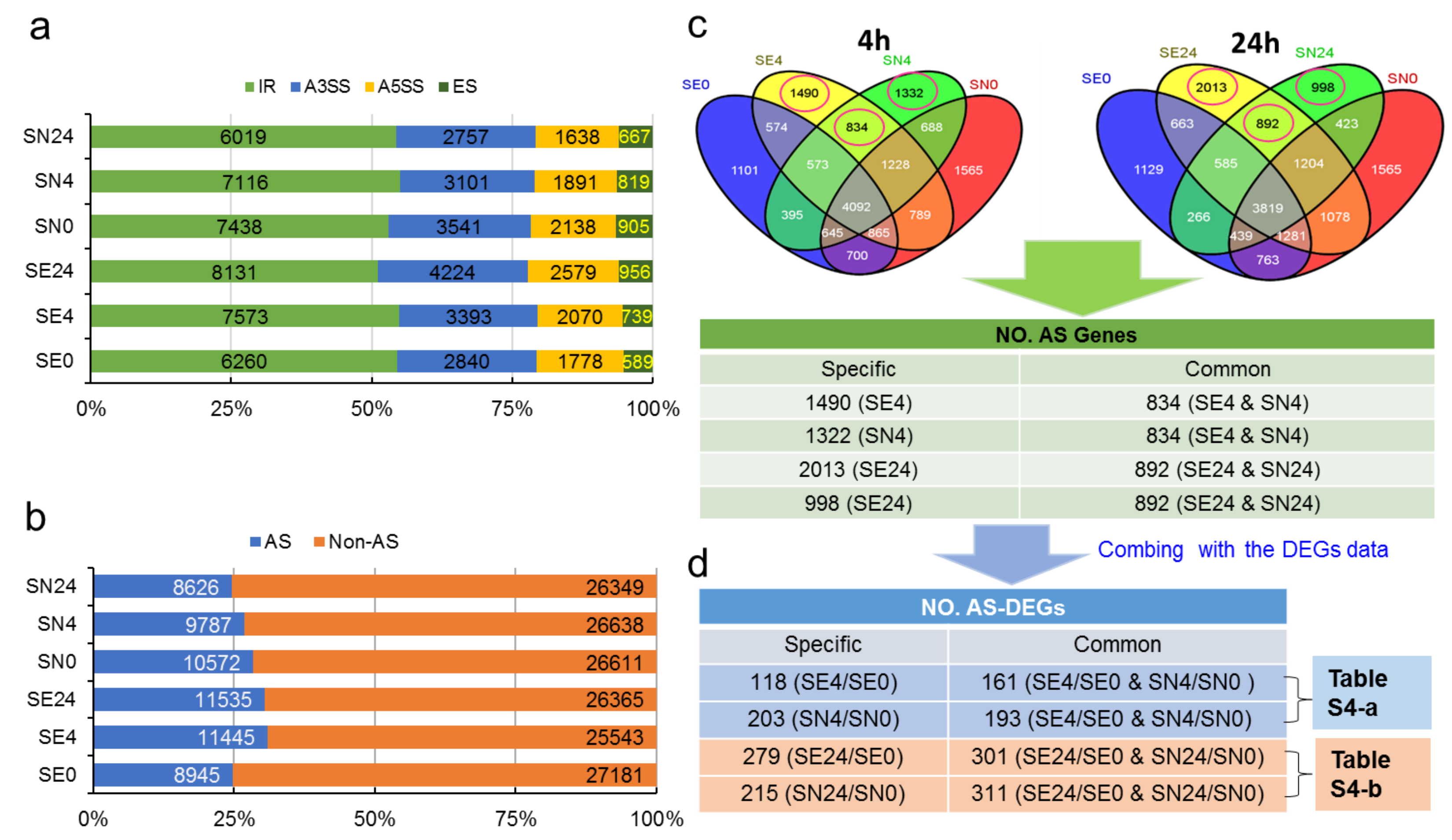

Figure S3 : Global comparison of alternative splicing events and genes.

a) The number and percent of intron retention, alternative 5' splice site, alternative 3' splice site and exon-skipping events under control and salt conditions in two genotypes.
b) The number and percent of alternative splicing genes and non-alternative splicing genes.
c) The number of specific and common AS genes in 4h or/and 24h of salt-treatment sample,
d) Combing all identified specific and common AS genes sets in 4h or/and 24h of salt-treatment sample with the corresponding DEGs data.

Sample	28 Known DE miRNA	Target	miRNA::Target	123 Novel DE miRNA	Target	miRNA::Target
E4/E0	12	85	163	53	593	710
E24/E0	17	165	273	62	623	808
N4/N0	2	8	16	40	413	502
N24/N0	11	34	61	48	424	475
Total	**28**	**209**	**349**	**106**	**929**	**1289**

Fig S4. The number of differentially expressed identified known and novel miRNAs in four comparisons. The differentially expressed identified known and novel miRNAs' targets predicted using psRNATarget web software online (http://plantgrn.noble.org/psRNATarget/)

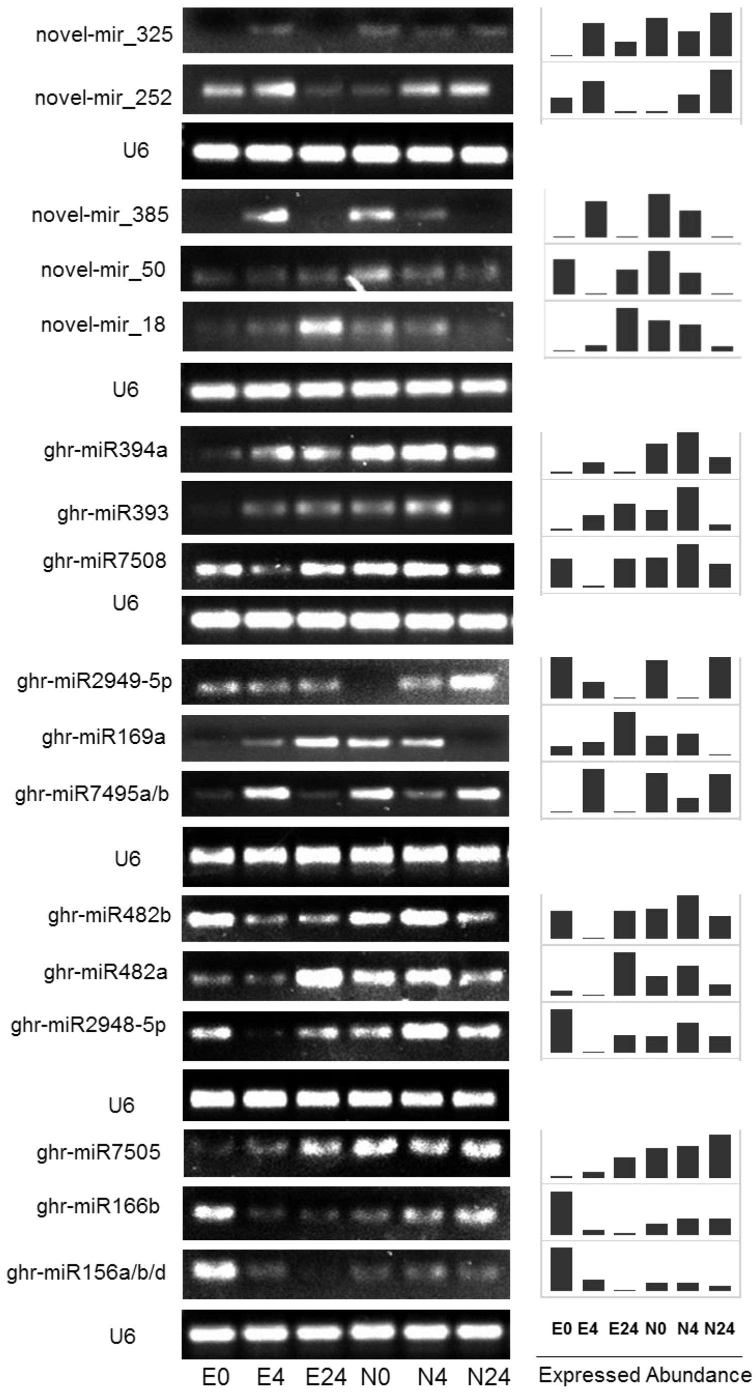

Figure S5: Expression profiles of 12 known and 5 novel miRNAs in two cotton genotypes from control (0) and salt-treated (4- and 24-h) samples by RT-PCR assays (left panel) and small RNA sequencing (right panel).

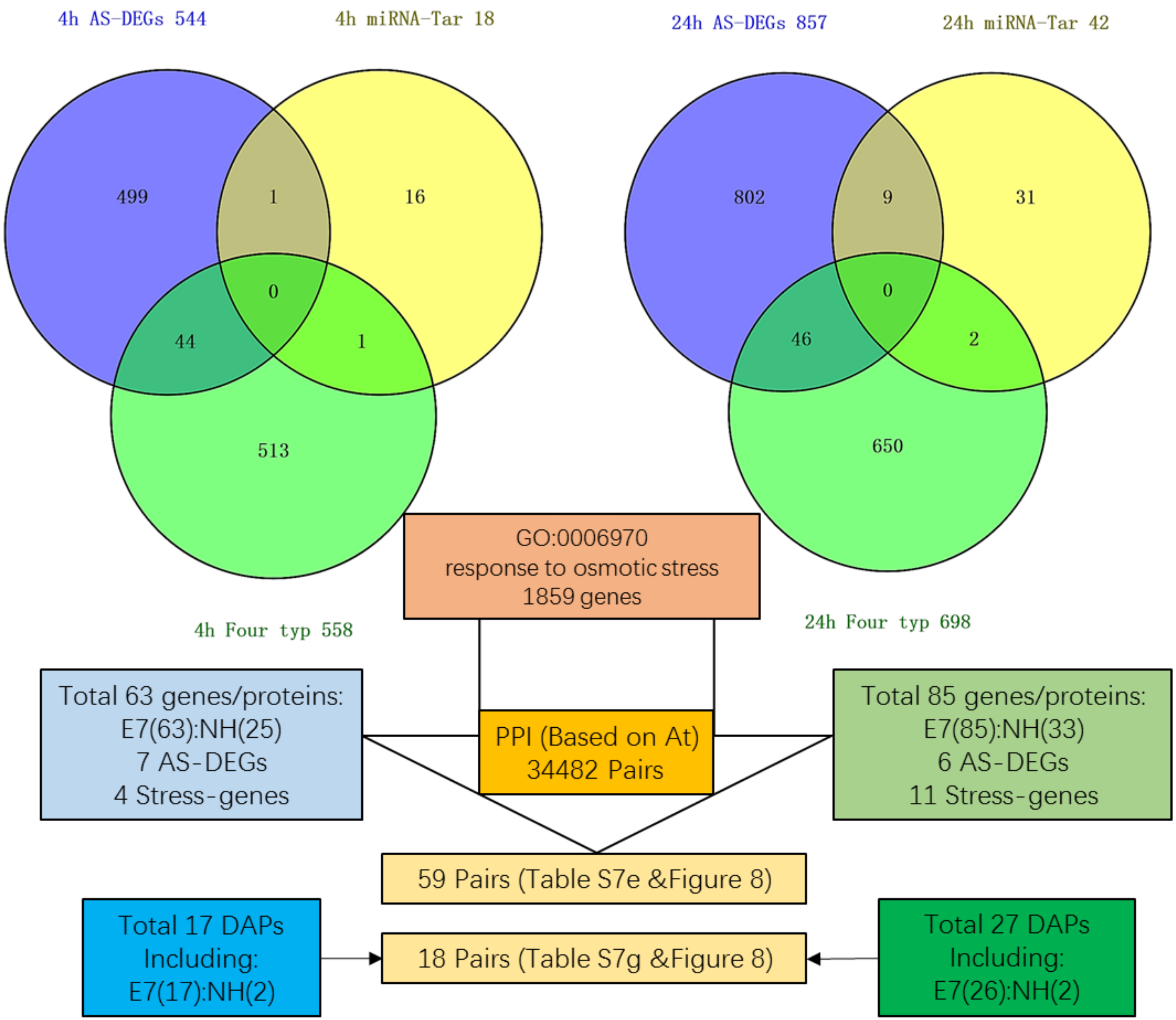

Figure S6: Flowchart analysis of the distinct genes/proteins between E7 and NH adapted to salt stress and protein–protein interaction.

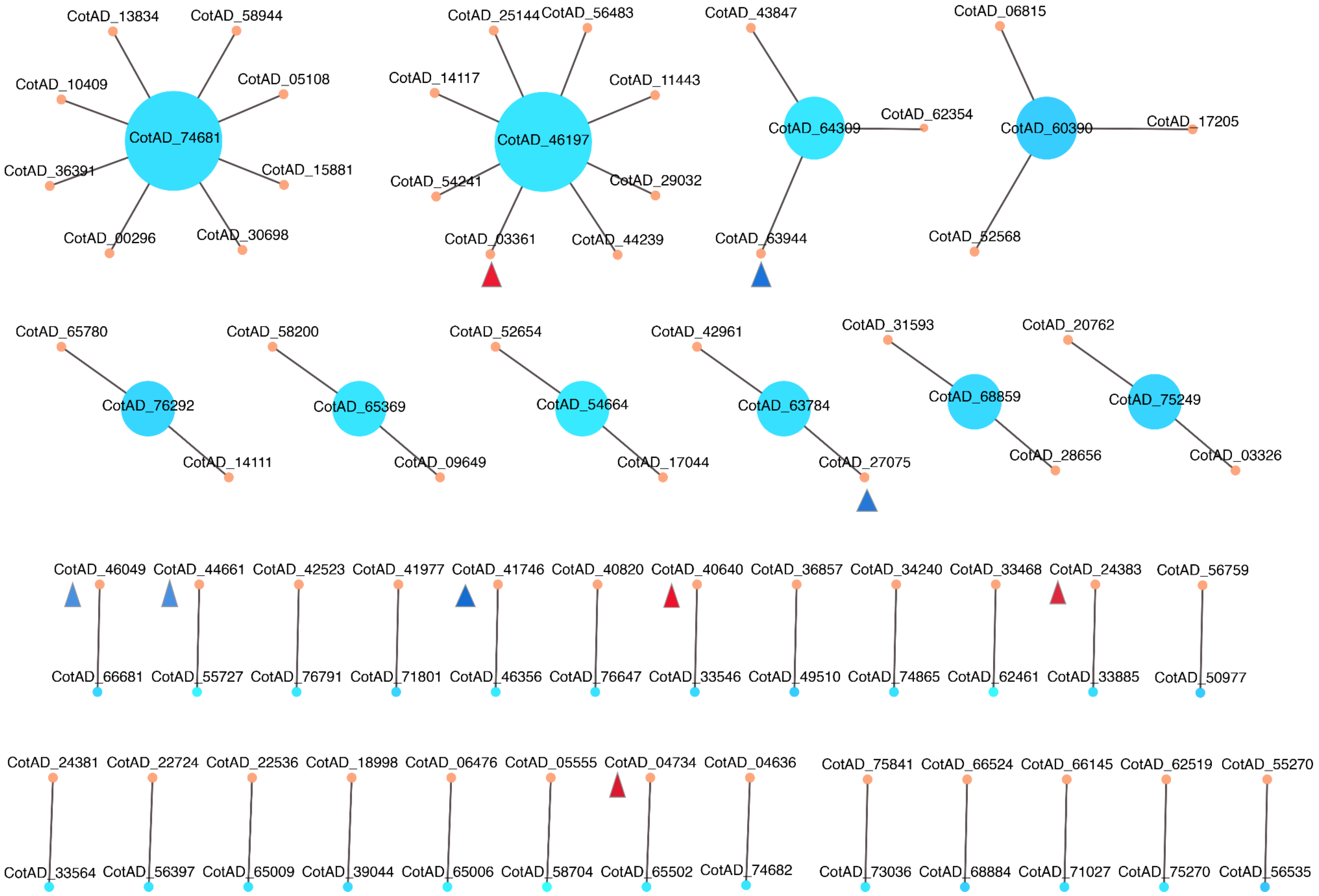

Figure S7: PPI network of the combination of genes and proteins that display contrasting expression patterns in the E7 and NH genotypes.The PPI interactions with a combined score greater than 160 in the STRING database were extracted to construct the network with Cytoscape version 3.5.0 (http://www.cytoscape.org/).
Orange boll represent DE transcripts or proteins, and blue coloring indicates the interactions of their target proteins;
The red and blue triangle represent the AS-DEGs in the E7 genotype after 4 h and 24 h of salt treatment, respectively.
